# Biochemical characterization of *Dimocarpus longan* polyphenol oxidase provides insights into its catalytic efficiency

**DOI:** 10.1038/s41598-022-20616-7

**Published:** 2022-11-25

**Authors:** Leela Ruckthong, Matthias Pretzler, Ioannis Kampatsikas, Annette Rompel

**Affiliations:** 1 Fakultät für Chemie, Institut für Biophysikalische Chemie, Universität Wien, Josef-Holaubek-Platz 2, 1090 Wien, Austria; 2 Faculty of Science, Department of Chemistry, King Mongkut’s University of Technology Thonburi (KMUTT), Thung Kru, Bangkok, 10140 Thailand

**Keywords:** Biochemistry, Bioinorganic chemistry, Metalloproteins, Chemistry, Biochemistry, Metalloproteins

## Abstract

The “dragon-eye” fruits produced by the tropical longan tree are rich in nutrients and antioxidants. They suffer from post-harvest enzymatic browning, a process for which mainly the polyphenol oxidase (PPO) family of enzymes is responsible. In this study, two cDNAs encoding the PPO have been cloned from leaves of *Dimocarpus longan (Dl)*, heterologously expressed in *Escherichia coli* and purified by affinity chromatography. The prepro-*Dl*PPO1 contains two signal peptides at its N-terminal end that facilitate transportation of the protein into the chloroplast stroma and to the thylakoid lumen. Removal of the two signal peptides from prepro-*Dl*PPO1 yields pro-*Dl*PPO1. The prepro-*Dl*PPO1 exhibited higher thermal tolerance than pro-*Dl*PPO1 (unfolding at 65 °C vs. 40 °C), suggesting that the signal peptide may stabilize the fold of *Dl*PPO1. *Dl*PPO1 can be classified as a tyrosinase because it accepts both monophenolic and diphenolic substrates. The pro-*Dl*PPO1 exhibited the highest specificity towards the natural diphenol (–)-epicatechin (k_cat_/K_M_ of 800 ± 120 s^−1^ mM^−1^), which is higher than for 4-methylcatechol (590 ± 99 s^−1^ mM^−1^), pyrogallol (70 ± 9.7 s^−1^ mM^−1^) and caffeic acid (4.3 ± 0.72 s^−1^ mM^−1^). The kinetic efficiencies of prepro-*Dl*PPO1 are 23, 36, 1.7 and 4.7-fold lower, respectively, than those observed with pro-*Dl*PPO1 for the four aforementioned diphenolic substrates. Additionally, docking studies showed that (–)-epicatechin has a lower binding energy than any other investigated substrate. Both kinetic and *in-silico* studies strongly suggest that (–)-epicatechin is a good substrate of *Dl*PPO1 and ascertain the affinity of PPOs towards specific flavonoid compounds.

## Introduction

Polyphenol oxidases (PPOs) are type-III dicopper metalloenzymes present in bacteria^1^, fungi^2,3^, archaea^4^, plants^5^, insects^6,7^, and animals including humans^8,9^. The PPO family contains tyrosinases (TYRs)^10–13^ and catechol oxidases (COs)^14,15^. TYRs catalyze the *ortho-*hydroxylation of monophenols to *o*-diphenols (monophenolase activity, EC 1.14.18.1) and subsequently the oxidation of the corresponding *o*-diphenols to *o*-quinones (diphenolase activity, EC 1.10.3.1), whereas COs can only perform the latter diphenolase activity^13,16^. The resulting quinones are highly reactive compounds and rapidly polymerize non-enzymatically, thereby forming yellow to black complex pigments known as melanins^17,18^.

In vivo, plant PPOs are translated as a precursor protein (prepro-PPO), corresponding to the translation of ~ 600 amino acids with approximately 60–75 kDa^19,20^. A prepro-PPO contains three distinct domains; an N-terminal signal sequence, the catalytically active domain that harbors the Cu-Cu site and a C-terminal domain shielding the active domain^5,20,21^ (Figure S1). The signal sequence (~ 80–100 amino acids) directs the protein to its sub-cellular destination while it is itself being cleaved off^22,23^. Following translocation across the thylakoid membrane the remaining passenger protein is processed to a latent form (pro-form) which is generally reported to be 45–69 kDa^14^. The binuclear copper center, where each copper is individually coordinated by three histidine residues^17,24^ located in the active domain, remains shielded by the C-terminal domain, effectively preventing the exposure of the active site to candidate substrates. The latency becomes lifted when the C-terminal domain is detached from the active site. In vitro, the disruption may be affected by proteases (e.g. trypsin, proteinase K)^25^, acidic or basic pH^24^, fatty acids^26^, and artificial detergents (e.g. sodium dodecyl sulfate, SDS)^17,27–33^. Although the natural proteolytic mechanism is unknown^17^, it has recently been reported that plant PPOs are capable of self-activation which can autoproteolytically separate the active enzyme from the C-terminal domain^34–36^.

Longan (*Dimocarpus longan, Dl*) belongs to the soapberry *Sapindaceae* family and is native to the subtropical Southeast Asia region^37,38^. The fruit is enriched with several nutritional components, especially the pericarp contains high amounts of phenolic compounds^39–41^ such as (–)-epicatechin, 4-methylcatechol, pyrogallol, caffeic acid, gallic acid, quercetin, vanillic acid and ferulic acid (Figure S2) that exhibit strong anti-inflammatory, antioxidant and anticancer activities^42,43^. Despite the health benefits obtained from the phenolic compounds, longan pericarp can deteriorate rapidly and develop a brown color two to three days after harvest under ambient temperature^44^. The presence of the resulting brown complexes substantially lowers the quality of the fruits. Physical approaches including fungicide dips, wax, chitosan coating, and sulfur fumigation have been applied to extend the storage life of longan^45–49^; however, the browning reaction has remained the main problem of the longan industry^47^. Though PPOs have been extensively investigated as the main cause of post-harvest browning in many agricultural products^11,12,50–61^, only few reports are available on the biochemical properties of longan PPO^62–64^. The enzyme was extracted from the natural source and was purified by ammonium sulfate precipitation and successive column chromatography (Sephadex G-200 and Phenyl Sepharose) or dialysis^62,63^. The optimal pH and temperature for the extracted longan PPO were reported to be 6.5 and 35 °C, respectively^62^. The enzyme was active towards pyrogallol, 4-methylcatechol and catechol. A further report also described (–)-epicatechin as the optimal endogenous substrate for longan^63^. A longan PPO gene was also cloned^64^ but has not been heterologously expressed. The enzyme classification of the longan PPOs remains unknown and their specificity and kinetic efficiency towards phenolic substrates have not been well-studied so far.

Here, two cDNAs containing PPO genes have been cloned from *Dimocarpus longan* leaves. The pro-*Dl*PPO1 encodes a latent form, while the prepro-*Dl*PPO1 encodes a precursor of the latent form that additionally contains an N-terminal signal sequence in its protein sequence. This signal sequence consists of a transit peptide which facilitates transport into the chloroplast stroma and a thylakoid-transfer signal that designates the thylakoid lumen as the protein’s final destination^65^. Usually, plant signal peptides are not recognized by bacteria. Although the whole sequence is translated into a protein, the fact that the signal peptides cannot be processed properly by bacteria can cause the whole protein to aggregate during expression. In this work a prepro-PPO has been successfully expressed in a prokaryotic system for the first time, was purified to homogeneity and biochemically analyzed in comparison to the analogous pro-enzyme. The kinetic properties of both variants towards phenolic substrates have been assessed. The kinetically characterized substrates were classified into two groups: natural substrates ((–)-epicatechin, 4-methylcatechol, caffeic acid and pyrogallol) (Figure S2) and standard substrates (tyramine, tyrosine, dopamine and *l*-DOPA) (Figure S3). Natural substrates refer to phenolic compounds that were previously detected in longan extracts by HPLC^39–41^ while standard substrates are those commonly used to characterized PPOs^3,17,66–68^ (which may or may not be present in longan fruits). Moreover, docking studies applying a model structure of *Dl*PPO1 were employed to investigate the enzyme–substrate interactions. The present study aimed at elucidating the biochemical impact of the presence of its signal sequence on *Dl*PPO1 and was designed to provide the fundamental information of substrate acceptance and specificity of *Dl*PPO1 that is necessary to devise new sustainable methods for ameliorating the post-harvest browning of this commercially important tropical fruit.

## Results and discussion

### Cloning, heterologous expression and purification of pro-*Dl*PPO1 and prepro-*Dl*PPO1

The PCR products on the complementary DNA of *D. longan* with primers enframing the ORF for prepro-*Dl*PPO1 and pro-*Dl*PPO1 yielded distinct bands of approximately 1800 bp and 1500 bp, respectively (Figure S4). After the cloning into the pGEX-6P-SG vector^69^, Sanger sequencing confirmed that the prepro-*Dl*PPO1 is composed of an ORF of 1797 nucleotides and encodes for 599 amino acids which are 100% identical to the amino acid sequence deduced from the published *Dl*PPO1 (Uniprot entry: A0A0K0NPU9)^64^. The pro-*Dl*PPO1 contains an ORF of 1512 nucleotides encoding 503 amino acids (Table S1) and has 99.60% identity with the previously published *Dl*PPO1 sequence^64^ resulting from five altered amino acids (Pro46Arg, Glu226Asp, Gln451His, Ile455Met, and Asn486Tyr, Figure S5). The pro-*Dl*PPO1 and prepro-*Dl*PPO1 enzymes were heterologously expressed in *E. coli* with an N-terminal glutathione-S-transferase tag from *Schistosoma japonicum*^69^ (see Materials and Methods). The temperatures upon the isopropyl-ß-D-1-thiogalactopyranoside (IPTG) induction during the expression were varied (37 °C, 28 °C and 17.5 °C) in order to investigate a condition favorable for high level production of soluble protein. Figure S6 shows that the most efficient expression temperature for both pro-*Dl*PPO1 and prepro-*Dl*PPO1 was at 17.5 °C, while 37 °C resulted in the lowest expression level of both soluble and non-soluble forms. Effective productions of soluble PPO enzymes under low temperature expressions were also reported for TYRs from *Juglans regia* (20 °C)^67^, *Agaricus bisporus* (20 °C)^3^, *Solanum lycopersicum* (20 °C)^17^, *Malus domestica* (20 °C)^66^, *Streptomyces avermitilis* (18 °C)^70^, *Streptomyces sp. ZL-24* (19 °C)^68^ and *Larrea tridentata* (25 °C)^32^.

The C-terminal domain of eukaryotic PPOs has been reported to be necessary for the folding of the catalytic domain^71^. So far, the expression of active grape PPO^72^ is the only example of successful production of a plant PPO without its C-terminal domain.

**Figure 1:**
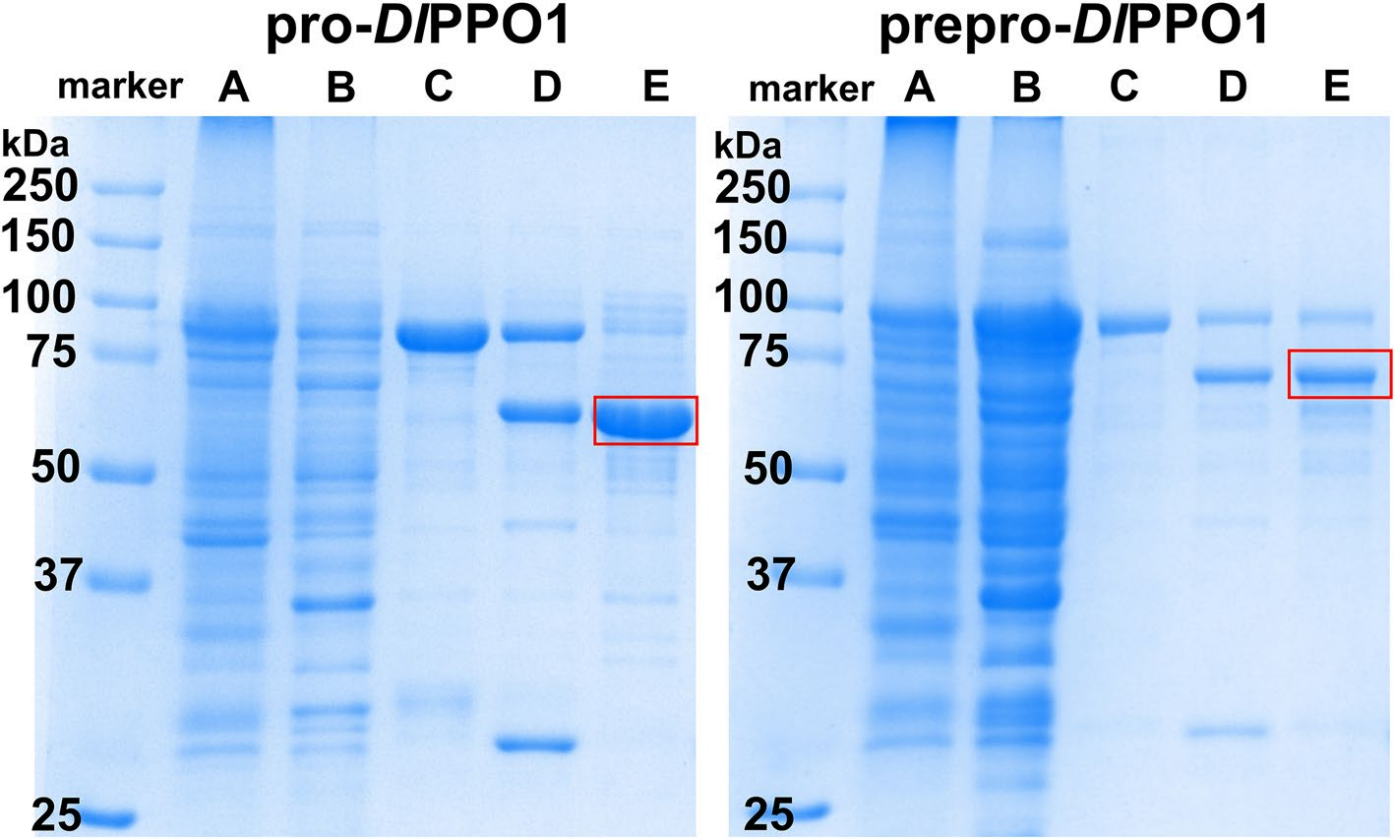
SDS-PAGE of pro-*Dl*PPO1 (left) and prepro-*Dl*PPO1 (right) at different stages of purification. (**A**) insoluble fraction of the bacterial cell pellets, (**B**) total soluble proteins, **C**) GST-*Dl*PPO1, which is the eluted GST-fusion protein after the glutathione affinity chromatography (first purification step on ÄKTA FPLC), (**D**) fractions of proteins after GST-*Dl*PPO1 was cut by HRV3C protease and **E**) final *Dl*PPO1 product (marked with a red frame; see Table 2) from the second round of glutathione affinity chromatography. The first lane of each gel is the molecular weight marker. The full-length gels are shown in Figure S8.

Through the purification by affinity chromatography applying the affinity of glutathione immobilized on cross-linked agarose, the expressions yielded 35.3 and 60.0 mg per liter of expression culture of GST-fused pro-*Dl*PPO1 and GST-fused prepro-*Dl*PPO1, respectively (Figure S7). Removal of the GST-fusion tag by the HRV3C protease resulted in 5.7 and 7.0 mg of the latent pro-*Dl*PPO1 and prepro-*Dl*PPO1, respectively (Table 1). The prepro-*Dl*PPO1 was successfully purified with 13.5% recovery of the total protein activity and a specific activity of 7.09 ± 0.05 U mg^−1^, while the pro-*Dl*PPO1 was retrieved with 19.6% recovery of the total protein activity and a specific activity of 28.3 ± 0.04 U mg^−1^. The copper contents of the *Dl*PPO1 variants were measured photometrically by reducing Cu(II) to Cu(I) with ascorbate and following the complexation of Cu(I) with 2,2′-biquinoline at 546 nm^73^. It was observed that the copper contents of pro-*Dl*PPO1 and prepro-*Dl*PPO1 were 1.3 ± 0.018 and 2.0 ± 0.030 copper ions per active site, respectively. The freshly purified proteins eluted in 50 mM Tris–HCl and 250 mM NaCl at pH 7.5 and were immediately exchanged to 100 mM MES and 200 mM NaCl at pH 6.5, and glycerol was applied at 30%(v/v) for storage of both forms at − 80 °C until use. The purity of the final pro-*Dl*PPO1 and prepro-*Dl*PPO1 preparations was confirmed by SDS-PAGE (Fig. 1 and Figure S8 for the full-length gels).

**Table 1:** Purification table for pro-*Dl*PPO1 and prepro-*Dl*PPO1 from 1 L of bacterial culture.

State of the enzyme	Purification step	Protein [mg]	Total activity [U]*	Specific activity [U mg^−1^] *	Yield [%]	Purification Fold
pro-*Dl*PPO1	Lysate	2730	819	0.300 ± 0.002	100.0	1.0
	GST-fusion protein	35.3	515	14.6 ± 0.02	63.0	48.7
	pro-*Dl*PPO1	5.7	161	28.3 ± 0.04	19.7	94.3
prepro-*Dl*PPO1	Lysate	4480	367	0.0820 ± 0.002	100.0	1.0
	GST-fusion protein	60.0	278	4.63 ± 0.30	75.7	56.5
	prepro-*Dl*PPO1	7.0	49.6	7.09 ± 0.05	13.5	86.5

*1 unit (U) is the amount of enzyme that catalyzes the conversion of 1 µmol of substrate per minute under the experimental conditions described in Materials and Methods. The enzymatic activity was measured with 8 mM of the monophenolic substrate tyramine and 0.25 mM SDS as activator.

### Molecular mass determination of pro-*Dl*PPO1 and prepro-*Dl*PPO1

**Figure 2:**
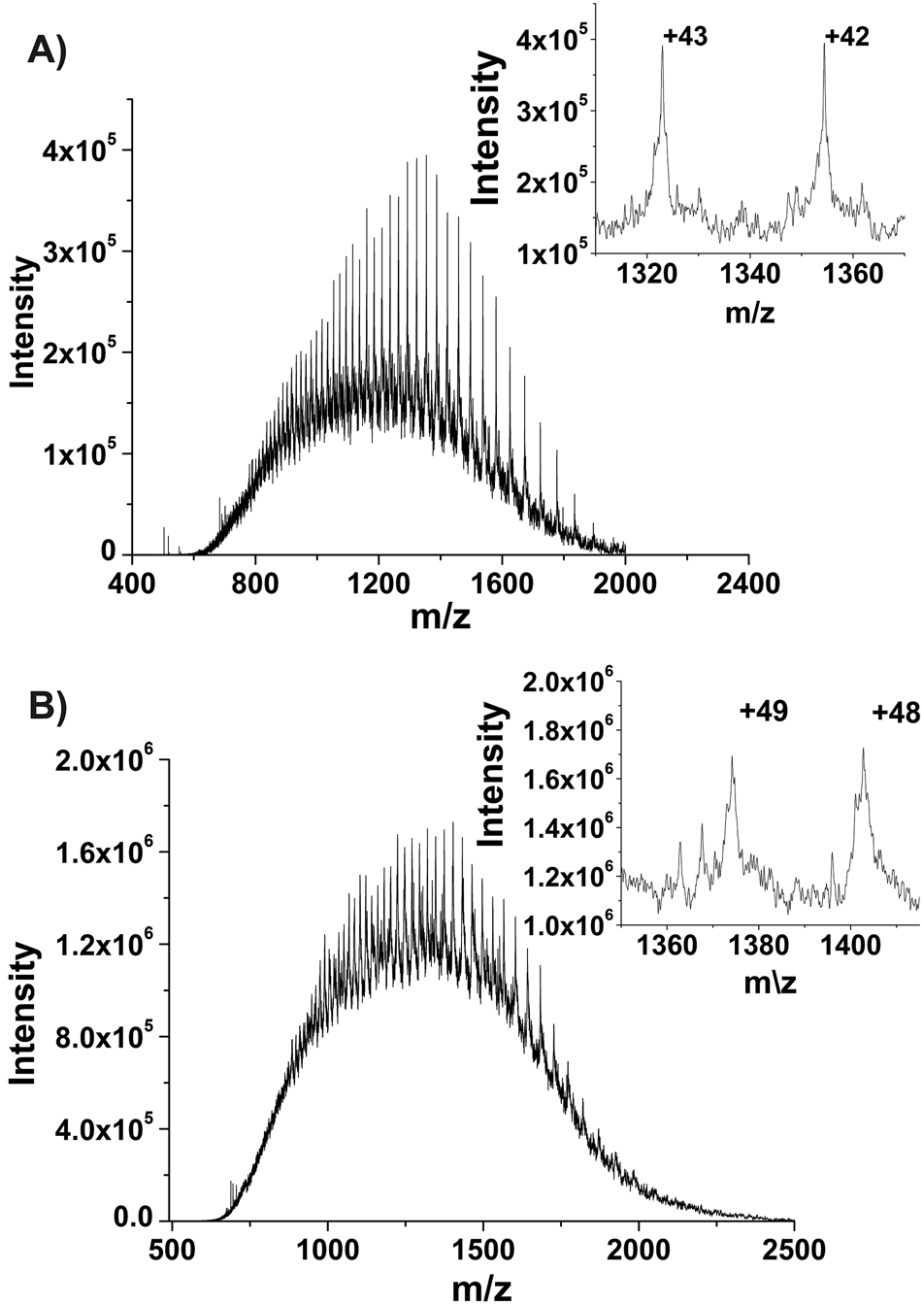
ESI–MS of the precursor prepro-*Dl*PPO1 and latent pro-*Dl*PPO1. The entire mass spectra of (**A**) pro-*Dl*PPO1 and (**B**) prepro-*Dl*PPO1 are shown, whereas the inset represents the magnified view of the two most prominent charge stages. The calculated and measured masses of the two states of the enzyme are listed in Table 2.

Gel electrophoresis demonstrated a prominent band of the final purified pro-*Dl*PPO1 and prepro-*Dl*PPO1 at approximately ~ 57 kDa and ~ 67 kDa, respectively, which suggested the latent (pro-*Dl*PPO1) and precursor (prepro-*Dl*PPO1) forms of longan PPO1 were obtained. The theoretical masses of pro-*Dl*PPO1 and prepro-*Dl*PPO1 were calculated from the respective protein’s sum formula using the atomic weight and isotopic composition of the constituent elements^74^ considering the presence of two conserved disulfide bonds (− 4H or − 4.032 Da) and one thioether bridge (− 2H or − 2.016 Da) as shown in Table 2. To confirm the actual molecular weights of the enzyme electrospray ionization—mass spectrometry (ESI–MS) was applied. The pro-*Dl*PPO1 yielded 56,845 Da (Fig. 2) which is in excellent agreement with the calculated mass of the protein starting from the vector-derived GlyProMet (marked as − 3, − 2, − 1 in Figure S5) up to the end of the latent PPO’s sequence (Ala 1 → Asp 503). This confirms that pro-*Dl*PPO1 was expressed as a latent PPO form with two disulfide bonds and one thioether bridge. In addition, prepro-*Dl*PPO1 was determined to have a mass of 67,285 Da, containing also one thioether bridge and two disulfide bonds (Fig. 2). The mass matches perfectly with the complete sequence of the prepro-polypeptide (Met 1 → Asp 599) plus the three amino acids GlyProMet (− 3, − 2, − 1) arising from the expression vector.

**Table 2:** Molecular masses of pro-*Dl*PPO1 and prepro-*Dl*PPO1, determined by ESI–MS.

PPO	Calculated M (-6H) [Da]	M (measured) [Da]	$\Delta /{\text{Da}}$
pro-*Dl*PPO1	56,844.05	56,844.92 ± 0.96	+ 0.87
prepro-*Dl*PPO1	67,284.68	67,285.04 ± 0.96	+ 0.37

### Thermal shift assay of pro-*Dl*PPO1 and prepro-*Dl*PPO1

**Figure 3:**
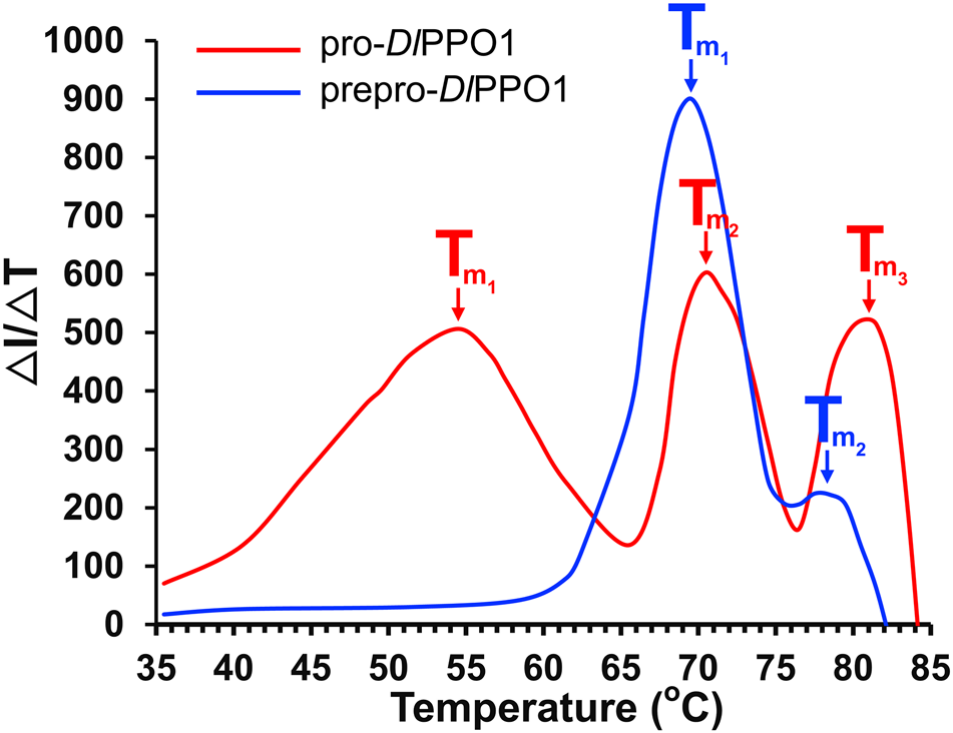
Thermal stability of the *Dl*PPO1 variants by thermal shift assays. The melting temperature values (T_m_) were determined from the derivative of fluorescence intensity (I, in arbitrary units) with respect to the temperature increment. The prepro-*Dl*PPO1 (blue line) resulted in T_m_ values of 69.5 °C and 77.5 °C. The pro-*Dl*PPO1 resulted in *T*_m_ values of 54.5 °C, 70.5 °C and 81.5 °C. The raw fluorescence intensities are shown in Figure S9.

The thermal shift assay has been used to assess the thermal stability of recombinant proteins^8,17,75^. Here, the thermal stability of the purified pro-*Dl*PPO1 and prepro-*Dl*PPO1 proteins at pH 6.5 (storage buffer condition) was tested. As shown in Fig. 3, for prepro-*Dl*PPO1 two melting temperatures (*T*_m_) at 69.5 °C and 77.5 °C were measured, while pro-*Dl*PPO1 exhibited three *T*_m_ values (54.5 °C, 70.5 °C, and 81.5 °C) until the complete denaturation of the enzymes. The unfolding of pro-*Dl*PPO1 began at much lower temperature (~ 40 °C) (Fig. 3, Figure S9) than for prepro-*Dl*PPO1 (~ 65 °C). The resulting first T_m_ of pro-*Dl*PPO1 is much lower than the other T_m_ values observed for both variants of the enzyme. This strongly indicates that the latent pro-*Dl*PPO1 exhibits some degree of limited unfolding at significantly lower temperature than its precursor prepro-*Dl*PPO1. As the pro-*Dl*PPO1 contains only domains that are also present in the prepro-*Dl*PPO1, it seems that the inclusion of the signal peptides does actually increase the overall thermal stability of *Dl*PPO1 and the signal peptides help to maintain protein stability.

### Activation of pro-*Dl*PPO1

#### Effect of SDS on pro-*Dl*PPO1 activity

The activation of pro-*Dl*PPO1 was monitored via the conversion of tyramine (Figure S3) to verify the proper SDS concentration for overcoming the enzyme’s latency. The reaction was examined using 0.5 µg of pro-*Dl*PPO1 and 8 mM tyramine as substrate in 50 mM MES at pH 7.0 with various SDS concentrations (0 to 1.5 mM). As displayed in Figure S10, an increase in SDS concentration from 0.00 to 0.25 mM caused a significant enhancement of the pro-*Dl*PPO1 activity, where the highest activation efficiency was achieved at 0.25 mM SDS (28.3 ± 0.04 U mg^−1^). At higher SDS concentrations, the enzymatic activity was gradually reduced. The loss in activity by half of the optimum was observed at an SDS concentration of 1.25 mM. Thus, the optimal SDS concentration for the activation of pro-*Dl*PPO1 was defined to be 0.25 mM and this level of added activator was applied in all subsequent experiments. A qualitatively identical activation behavior was seen for prepro-*Dl*PPO1 (Figure S10).

Most enzymes lose their biological activities under treatment with SDS, while PPOs are resistant to SDS to some extent, probably in part due to the existence of disulfide bonds in the structure^55,76,77^. SDS initiates the activation of PPOs by introducing conformational changes of the N- and C-termini of the latent form which allow for exposure of the active site and promote substrate access^78^. PPO activation is generally achieved at low SDS concentrations, while higher concentrations of the anionic detergent significantly inhibit the enzymatic activity^79,80^, which is consistent with the SDS activation profile observed for *Dl*PPO1. The use of SDS for the activation of latent PPOs has been reported to be the best activation method in vitro as it almost completely eliminates the latency of plant PPOs and provides an activity closely resembling mature PPO (after the removal of the C-terminal domain)^66^. The degree of SDS activation varies greatly with plant extracts^78^; for examples, 0.13 mM SDS was required to activate latent potato PPO (*Solanum tuberosum*)^33^, 0.35 mM SDS for mushroom PPO (*Agaricus bisporus*)^81^, 0.69 mM SDS for table beet PPO (*Beta vulgaris*)^82^, 2 mM SDS for peach PPO (*Prunus persica* cv. Paraguaya)^83^ and 6.93 mM SDS for mango PPO (*Mangifera indica*)^84^. The necessary concentration of detergent for activation of dandelion PPO has been shown to be determined primarily by the amino acids forming the interface between the catalytic and the C-terminal domain^85^. The use of 0.25 mM SDS for the pro-*Dl*PPO1 activation is at the lower end when compared to other heterologously expressed PPOs such as walnut *Jr*PPO1 (2 mM SDS)^67^, tomato *Sl*PPO1 and *Sl*PPO2 (1.5 mM SDS)^17^, mushroom *Ab*PPO4 (2 mM SDS)^3^ and for apple *Md*PPO1 (3 mM), *Md*PPO2 (2 mM) and *Md*PPO3 (4 mM)^66^.

#### Effect of pH on the pro-*Dl*PPO1 activity

The pH is one of the parameters that affects a PPO’s activity^86^. To optimize the pH of the reaction buffer for the pro-*Dl*PPO1 activity, the enzyme reaction was examined through 50 mM MES and TRIS buffers ranging from pH 5.5 to 8.5 using 0.5 µg of pro-*Dl*PPO1, 8 mM tyramine as substrate and 0.25 mM SDS for activation of the enzyme. Figure S11 demonstrates that the enzyme shows different degrees of activity in this pH range. The highest activity was exhibited at pH 7.0 (28.3 ± 0.04 U mg^−1^) in MES buffer whilst the activity slightly decreased at pH 6.5 by 17% and was significantly reduced at pH 6.0 and 5.5 by 61% and 86%, respectively. Likewise, the activity significantly dropped at higher pH (pH 7.5–8.5). A decrease by 43% was observed at pH 7.5 and by 74% at pH 8.0. The activity was almost depleted at pH 8.5 with a 96% activity loss from the optimum. A pH 7.0 optimum was previously reported for the tyrosinases in apple (*Md*PPO1 and *Md*PPO3)^66^ and mushroom (*Ab*PPO4)^3^. A slightly higher optimal pH at 7.5 was observed in *Md*PPO3^66^. *Jr*PPO1 and *Jr*PPO2 from walnut had lower pH optima of pH 6.0^67^, whereas the bacterial tyrosinase from a *Streptomyces sp.* from peatland (*Sz*TYR) revealed an unusually high pH optimum of pH 9.0 as a result of adaptation to its natural pH environment^68^.

#### Substrate specificity and enzyme kinetics

The catalytic performance of the pro-*Dl*PPO1 was first examined using four standard substrates (Figure S3): two monophenols (tyramine, *d*-and *l*-tyrosine) and two diphenols (dopamine and *l*-DOPA) were applied at the enzyme’s optimal pH 7.0 and an SDS concentration of 0.25 mM. The pro-*Dl*PPO1 does accept all four standard substrates from the monophenolic and diphenolic categories, and consequently can be classified as a TYR (EC 1.14.18.1). K_M_ and k_cat_ values were calculated by nonlinear regression and the substrate specificity was estimated using the k_cat_/K_M_ ratio. Data reported on the substrate specificity for pro-*Dl*PPO1 are shown in Table 3 and Figure S13. The diphenolic substrate dopamine is processed faster (k_cat_: 260 ± 22 s^−1^) than *l*-DOPA (k_cat_: 98 ± 8.5 s^−1^), and the monophenol tyramine (k_cat_: 35 ± 1.9 s^−1^) is hydroxylated faster than *l-* or *d*-tyrosine. Moreover, pro-*Dl*PPO1 has a lower K_M_ value for dopamine (K_M_: 2.0 ± 0.35 mM), thus resulting in the highest catalytic efficiency (k_cat_/K_M_) among the four tested standard substrates. The k_cat_/K_M_ ratio for dopamine is 4.6-, 16-, 336- and 504-fold higher than that of *l*-DOPA (28 ± 3.9 s^−1^ mM^−1^), tyramine (8.2 ± 0.91 s^−1^ mM^−1^), *d*-tyrosine (0.39 ± 0.016 s^−1^ mM^−1^) and *l*-tyrosine (0.26 ± 0.016 s^−1^ mM^−1^), respectively (Table 3). The prepro-*Dl*PPO1 also accepts both standard monophenols (tyramine and tyrosine) and diphenols (dopamine and *l*-DOPA), but with different degrees of catalytic efficiency (Table 3 and Figure S14). The prepro-*Dl*PPO1 binds dopamine with a similar K_M_ value (K_M_: 2.3 ± 0.27 mM) to that observed for the pro form (K_M_: 2.0 ± 0.35 mM); however, the catalytic efficiency of prepro-*Dl*PPO1 is reduced 7.5-fold compared to pro-*Dl*PPO1 with a k_cat_/K_M_ of 17 ± 2.4 s^−1^ mM^−1^. The conversion of the diphenol *l*-DOPA by prepro-*Dl*PPO1 (k_cat_/K_M_ of 12 ± 1.1 s^−1^ mM^−1^) was 2.4-fold slower due to the lower catalytic activity (k_cat_: 40 ± 2.1 s^−1^) compared to pro-*Dl*PPO1. A similar observation was noted for the monophenolic substrates: The hydroxylation of tyramine (k_cat_/K_M_ of 1.6 ± 0.14 s^−1^ mM^−1^) by prepro-*Dl*PPO1 is 5.2-fold less efficient than for pro-*Dl*PPO1 with a concomitant increase of K_M_ to 15 ± 1.0 mM and a reduced catalytic activity (k_cat_: 24 ± 1.4 s^−1^). The kinetic examination towards the monophenolics *l-* and *d*-tyrosine demonstrated decreased k_cat_/K_M_ ratios, which are 1.3- and 1.6-fold lower than those observed for pro-*Dl*PPO.

**Table 3:** Kinetic parameters of pro-*Dl*PPO1 and prepro-*Dl*PPO1.

Enzyme	Substrate	K_M_ (mM)	k_cat_ (s^−1^)	k_cat_/K_M_ (s^−1^ mM)
pro-*Dl*PPO1	tyramine	4.3 ± 0.42	35 ± 1.9	8.2 ± 0.91
	*l*-tyrosine	**	**	0.26 ± 0.016
	*d*-tyrosine	**	**	0.39 ± 0.016
	*l*-DOPA	3.4 ± 0.37	98 ± 8.5	28 ± 3.9
	dopamine	2.0 ± 0.35	260 ± 22	130 ± 25
	(–)-epicatechin	0.35 ± 0.052	270 ± 15	800 ± 120
	4-methylcatechol	0.57 ± 0.091	330 ± 17	590 ± 99
	caffeic acid	0.70 ± 0.11	3.0 ± 0.19	4.3 ± 0.72
	pyrogallol	2.7 ± 0.34	190 ± 11	70 ± 9.7
	gallic acid	No activity		
	quercetin			
	vanillic acid			
	ferulic acid			
prepro-*Dl*PPO1	tyramine	15 ± 1.0	24 ± 1.4	1.6 ± 0.14
	*l*-tyrosine	**	**	0.20 ± 0.032
	*d*-tyrosine	**	**	0.24 ± 0.036
	*l*-DOPA	3.3 ± 0.26	40 ± 2.1	12 ± 1.1
	dopamine	2.3 ± 0.27	40 ± 2.4	17 ± 2.4
	(–)-epicatechin	1.1 ± 0.12	36 ± 2.0	33 ± 4.1
	4-methylcatechol	4.4 ± 0.37	73 ± 4.0	17 ± 1.7
	caffeic acid	0.71 ± 0.046	1.76 ± 0.084	2.5 ± 0.20
	pyrogallol	4.1 ± 0.28	61 ± 3.0	15 ± 1.2
	gallic acid	No activity		
	quercetin			
	vanillic acid			
	ferulic acid			

**The substrate solubilities were too low for a determination of the kinetic parameters using the Michaelis–Menten model. Linearization was subsequently implemented considering substrate concentration <  < K_M_, yielding k_cat_/K_M_ for *l*- and *d*-tyrosine as displayed in the Table.

To gain insights into the kinetic behavior of *Dl*PPO1 in its natural environment, a series of natural phenolic substrates ((–)-epicatechin, 4-methylcatechol, caffeic acid, pyrogallol, gallic acid, quercetin, vanillic acid and ferulic acid (Figure S2)), all of which have been detected in longan peel, pulp and seed^39–41^, were assessed. Data reporting the natural substrate specificity of pro-*Dl*PPO1 are shown in Table 3 and Figure S15. Through the kinetic studies it was revealed that pro-*Dl*PPO1 is active towards the polyphenols (–)-epicatechin, 4-metylcatechol, caffeic acid and pyrogallol, while the enzyme was completely inactive with the rest of the tested substrates. The highest efficiency of pro-*Dl*PPO1 was observed towards (–)-epicatechin (k_cat_: 270 ± 15 s^−1^) with a K_M_ of 0.35 ± 0.052 mM, resulting in k_cat_/K_M_ of 800 ± 120 s^−1^ mM^−1^. This evidence suggests that (–)-epicatechin is highly likely to be one of the physiological substrates of *Dl*PPO1 in longan. The rates of 4-methylcatechol, pyrogallol and caffeic acid conversions were lower than for (–)-epicatechin with resulting k_cat_/K_M_ values of 590 ± 99 s^−1^ mM^−1^, 70 ± 9.7 s^−1^ mM^−1^ and 4.3 ± 0.72 s^−1^ mM^−1^, respectively. However, the catalytic efficiency towards these natural substrates significantly decreased in the prepro-*Dl*PPO1. The kinetic examination with (–)-epicatechin resulted in a higher K_M_ value (1.1 ± 0.12 mM), indicating that the precursor has lower specificity towards this substrate than pro-*Dl*PPO1 (K_M_ = 0.35 ± 0.052 mM). Thus, the catalytic efficiency of prepro-*Dl*PPO1 was observed to be only 33 ± 4.1 s^−1^ mM^−1^, which is 23-fold lower than the value of pro-*Dl*PPO1. Likewise, the specificity of the prepro-*Dl*PPO1 to 4-methylcatechol, caffeic acid and pyrogallol is less than the pro-*Dl*PPO1 as indicated by the higher K_M_ values in comparison to the pro-*Dl*PPO1. Data reporting the natural substrate specificity of prepro-*Dl*PPO1 are shown in Table 3 and Figure S16. The resulting k_cat_/K_M_ values of 17 ± 1.7 s^−1^ mM^−1^ (4-methylcatechol), 2.5 ± 0.20 s^−1^ mM^−1^ (caffeic acid) and 15 ± 1.2 s^−1^ mM^−1^ (pyrogallol) indicate that the catalytic efficiency of the prepro-*Dl*PPO1 decreased 36, 1.7 and 4.7-fold, respectively, from the pro-*Dl*PPO1. Thus, the signal peptide component could be one of the factors responsible for the reduced rate of PPOs in catalyzing the oxidation and hydroxylation of phenolic compounds.

With concentrations of (–)-epicatechin in excess of 2 mM the enzymatic conversion was slower than the rate seen at lower substrate levels for both prepro-*Dl*PPO1 and pro-*Dl*PPO1 (Figures S15A and S16A). Including competitive inhibition by the substrate itself (“substrate inhibition”, Equation S3) in the Michaelis–Menten model allowed to expand the modelled concentration range up to 3 mM for pro-*Dl*PPO1 and up to 5 mM for prepro-*Dl*PPO1. Changing the mathematical model of enzymatic conversion causes major changes to the obtained kinetic parameters. For pro-*Dl*PPO1 (K_M_ = 4.6 ± 2.0 mM, k_cat_ = 1500 ± 590 s^−1^, K_i_ = 0.56 ± 0.26 mM) as well as for prepro-*Dl*PPO1 (K_M_ = 6.1 ± 3.7 mM, k_cat_ = 140 ± 72 s^−1^, K_i_ = 1.2 ± 0.79 mM) both K_M_ and k_cat_ are significantly increased (compare Table 3) to compensate for the newly introduced inhibiting effect of the substrate itself (K_i_) while the variances of all fitted parameters are seriously inflated relative to the simple Michaelis–Menten model. For pro-*Dl*PPO1 and dopamine (Figure S13C; K_M_ = 1.2 ± 0.11 mM, k_cat_ = 250 ± 15 s^−1^, K_i_ = 30 ± 7.3 mM), with a much higher K_i_ indicating less efficient substrate inhibition, the inflation of the parameter’s variances is less severe and both k_cat_ and K_m_ are slightly lower than with the simple Michaelis–Menten model.

The binuclear copper center of PPOs was reported to function enantioselectively^32^. The enantioselectivity of the catalytic reaction of pro-*Dl*PPO1 on tyrosine was investigated. The enzyme showed a preference for *d*-tyrosine with a slightly higher catalytic efficiency of 0.39 ± 0.016 s^−1^ mM^−1^ over the *l*-enantiomer (0.26 ± 0.016 s^−1^ mM^−1^). 1 mM of *d*-tyrosine is converted at a rate of 0.11 ± 0.01 U mg^−1^, which is 1.1-fold faster than the rate of *l*-tyrosine conversion by pro-*Dl*PPO1. The effect of substrate chirality was also observed in mushroom *Ab*PPO4 where *l*-tyrosine was slightly more preferable than the *d-*counterpart^3^. The tyrosinase produced by *Streptomyces sp. REN-21* reacted 35.9-fold faster on *l*-tyrosine than *d*-tyrosine, which is the strongest enantioselectivity effect on the substrate tyrosine among PPOs that has been reported^87^. The preference towards a *d*-stereoisomer substrate was also reported in a PPO purified from eggplant (*Solanum melongena*), in which the conversion of 0.1 mM *d*-DOPA was 1.8-fold faster than *l*-DOPA^88^. Another example of the enantiospecific reaction in plant PPOs is found in larreatricin hydroxylase from *Larrea tridendata* (*Lt*PPO) which exhibits a pronounced preference (23-fold) for ( +)-larreatricin in comparison to the (–)-enantiomer, whereas in the same study *Ab*PPO4 reveals an opposite effect by preferring (–)-larreatricin (13-fold)^32^.

#### Formation of the *oxy*-form in prepro-*Dl*PPO1 and pro-*Dl*PPO1

**Figure 4:**
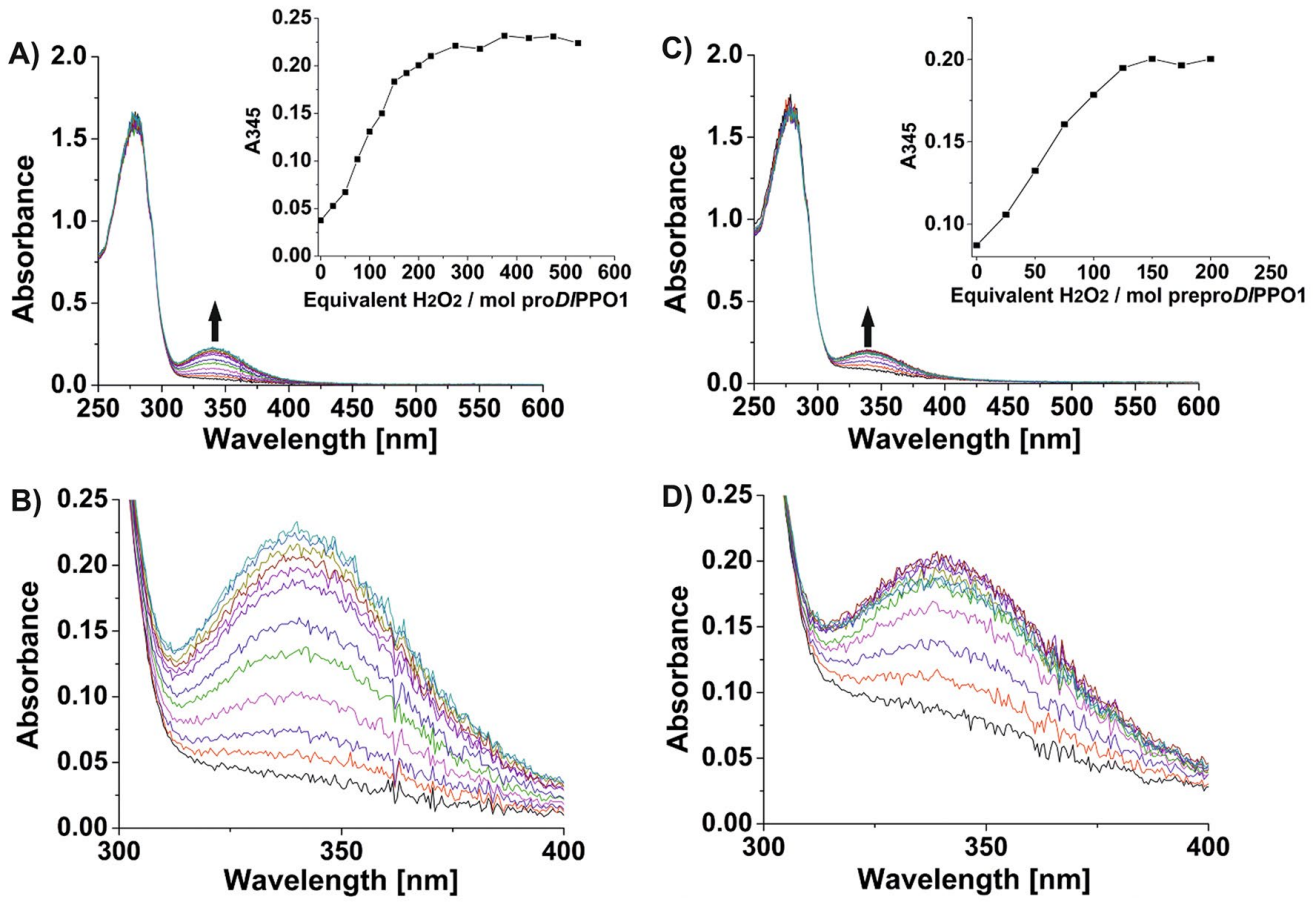
UV/Vis spectra of pro-*Dl*PPO1 and prepro-*Dl*PPO1 after treatment with H_2_O_2_. (**A**) Overall spectra of pro-*Dl*PPO1 after treatment with H_2_O_2_. The inset shows the absorption at 345 nm vs. the added equivalents of H_2_O_2_. (**B**) Overall spectra of prepro-*Dl*PPO1 after treatment with H_2_O_2_. The inset shows the absorption at 345 nm vs. equivalents of H_2_O_2_. (**C**) Zoomed-in view of the spectra at 300–400 nm of the pro-*Dl*PPO1 after treatment with H_2_O_2_. (**D**) Close-up view of the spectra at 300–400 nm of the prepro-*Dl*PPO1 after treatment with H_2_O_2_.

The prepro-*Dl*PPO1 and pro-*Dl*PPO1 were spectrophotometrically examined using H_2_O_2_ to assess the formation of the *oxy*-state of the enzyme in the presence of oxygen. Addition of H_2_O_2_ to the prepro-*Dl*PPO1 leads to an increase of the specific absorption band (~ 345 nm), which is indicative of the oxygen-induced *oxy*-form characteristic for type-III copper center enzymes and has been attributed to the charge transfer transition of ${\text{O}}_{2}^{2 - } \left( {\pi_{\sigma }^{*} } \right) \to {\text{Cu}}\left( {II} \right)_{{d_{{x^{2} - y^{2} }} }}$^89^. A similar observation was made with pro-*Dl*PPO1, where the *oxy* adduct formation resulted in the formation of a pronounced absorption band at 345 nm (Fig. 4). The absorption at 345 nm became saturated when the titration reached ~ 150 equivalents of H_2_O_2_ for prepro-*Dl*PPO1 and ~ 175 equivalents of H_2_O_2_ for pro-*Dl*PPO1, respectively. The resulting absorption coefficients are ~ 9000 M^−1^ cm^−1^ per mol enzyme for the prepro- *Dl*PPO1 and ~ 10,300 M^−1^ cm^−1^ per mol enzyme for the pro-*Dl*PPO1. The formation of the *oxy*-form by H_2_O_2_ has previously been shown for PPOs extracted from natural sources^60,90,91^. Catechol oxidases purified from lemon balm (*Melissa officinalis*)^86^, sweet potato (*Ipomoea batatas*)^87^ and walnut leaves (*Juglans regia*)^52^ resulted in a full saturation of the *oxy* form when two equivalents of H_2_O_2_ were added. In gypsywort (*Lycopus europaeus*) catechol oxidase, 6 equivalents of H_2_O_2_ were required to saturate the λ_345_ band, while in black poplar (*Populus nigra*)^89^ 80 equivalents of H_2_O_2_ were needed. Heterologously expressed *Sl*PPO1 and *Sl*PPO2 from *Solanum lycopersicum*^17^ were reported to generate the fully saturated *oxy* form at 25 and 11 equivalents of H_2_O_2_ addition, respectively. Moreover, the addition of H_2_O_2_ induced the wildtype *Cg*AUS from *Coreopsis grandiflora* that was heterologously expressed in *E. coli* to fully form an *oxy*-adduct at 24 equivalents of H_2_O_2_^92,93^. The present study is the first to investigate the *oxy*-form formation of a heterologously expressed prepro-PPO which still has its signal peptides attached.

#### Molecular docking simulations of *Dl*PPO1

Molecular docking studies were performed to provide more insight into the enzyme–substrate interactions of the pro-*Dl*PPO1. The simulation studies were carried out using the AutoDock Vina software^94^. The structural model of pro-*Dl*PPO1 was prepared as described in Materials and Methods and was used for the computational calculations. Binding poses were calculated for all the investigated substrates (Figures S17 and S18) and validated according to the TYR structure of *Bacillus megaterium* which contains the substrate *l*-tyrosine bound to the Zn-substituted active site (PDB: 4P6R)^1^. The results provided detailed information about the putative position of the investigated substrates and the binding energies of these poses around the active center (Table S3). The natural substrate (–)-epicatechin exhibited the strongest binding affinity among the investigated substrates as strongly suggested by the lowest measured K_M_ (K_M_ = 0.35 ± 0.052 mM) and the most favorable binding energy (Table S3). The higher catalytic efficiency in combination with the similar K_M_ values of 4-methylcatechol and caffeic acid indicates that 4-methylcatechol forms a more stable complex with *Dl*PPO1 than caffeic acid does. Pyrogallol features the highest K_M_ among the tested natural substrates, suggesting weak binding interactions between pro-*Dl*PPO1 and the substrate. The higher catalytic efficiency than the one observed for caffeic acid is due to the much higher k_cat_ value of pyrogallol (Table 3).

**Figure 5:**
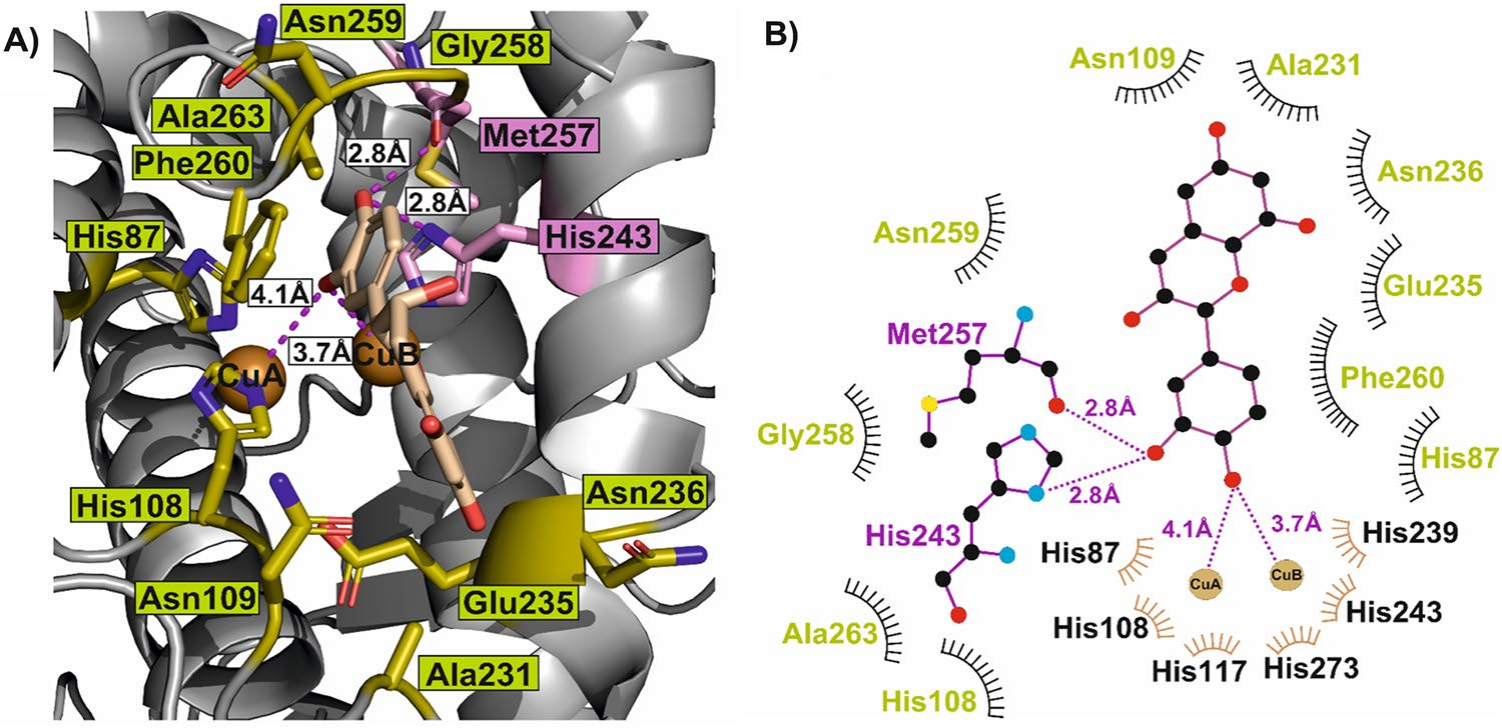
Docking of (–)-epicatechin to *Dl*PPO1. (**A**) Binding pose of (–)-epicatechin in the active center of *Dl*PPO1. (**B**) *Dl*PPO1 and (–)-epicatechin interaction plot. Color codes: Magenta text represents residues forming hydrogen bonds and olive text marks residues exhibiting hydrophobic interactions. In (–)-epicatechin as well as in the two amino acids that form hydrogen bonds, carbon atoms are shown as black filled circles, oxygen atoms are drawn red, nitrogen atoms are shown in blue and the single sulfur atom present in the interaction plot is highlighted in orange.

Further analysis with LiqPlot + of (–)-epicatechin docked to the active center of the *Dl*PPO1 revealed the specific interactions of the substrate with the amino acids around the dicopper active center. Specifically, one of the two hydroxy groups on the (–)-epicatechin’s *o*-diphenolic ring (Fig. 5) establishes two hydrogen bonds; one with the imidazole nitrogen of the conserved copper-coordinating His243 (2.8 Å) and the second one with the carboxylic backbone group of Met257 (2.8 Å). The second hydroxy group of the same ring interacts with the two copper ions and is well-positioned for the oxidative reaction (CuA: 4.1 Å and CuB: 3.7 Å, respectively). Moreover, nine hydrophobic interactions with the residues His87, Asn109, Ala231, Glu235, Asn236, Gly258, Asn259, Phe260 and Ala263 cover the whole body of the substrate (Fig. 5). Similar to other plant PPOs the conserved Phe260^17^, the gatekeeper residue, turns to the phenolic ring of the (–)-epicatechin and forms sandwich type *π-π* interactions. The high affinity binding of (–)-epicatechin showcases the preference of plant PPOs for phenolic secondary metabolites and strongly suggests (–)-epicatechin to be a physiological substrate of *Dl*PPO1. Similar results have been presented for the tomato PPO (*Sl*PPO1) which has a high affinity towards the dihydrochalcone phloretin^17^ and for the aurone synthase from *Coreopsis grandiflora* for which butein was proposed as its natural substrate^51,95^.

## Conclusion

In this work, prepro-*Dl*PPO1 from *D. longan* was expressed, purified and characterized in comparison with pro-*Dl*PPO1. This constitutes the first report on the biochemical differences between a PPO expressed in a prokaryotic system both with and without its N-terminal signal sequence. Titration with H_2_O_2_ monitored by UV–Vis spectroscopy verified that *Dl*PPO1 contains a type-III copper center. After addition of ~ 150 and ~ 175 equivalents of H_2_O_2_, the absorption band around 345 nm indicating the *oxy*-form was fully developed in prepro-*Dl*PPO1 and pro-*Dl*PPO1, respectively (Fig. 4). Kinetic experiments showed that both enzymes accept monophenolic substrates, both forms of *Dl*PPO1 were thus classified as tyrosinases (EC. 1.14.18.1). The pro-*Dl*PPO1 exhibited lower K_M_ and higher k_cat_ values, and consequently much higher kinetic efficiencies, towards all tested substrates than prepro-*Dl*PPO1 did (Table 3). The N-terminal signal peptides seem to provide an efficient barrier for substrate binding to the active site of *Dl*PPO1. Inclusion of the supposedly unfolded signal peptides resulted in a substantial thermal tolerance of prepro-*Dl*PPO1 that even surpasses the thermal stability of pro-*Dl*PPO1 (Fig. 3). In combination with the signal peptides’ impeding effect on the catalytic function of *Dl*PPO1, the increased thermostability they confer to *Dl*PPO1 may indicate a more ordered and compact structure of these peptides as currently envisioned. (–)-Epicatechin exhibited the lowest K_M_, the highest catalytic efficiency (Table 3), the most favorable binding to *Dl*PPO1 (Table S3 and Fig. 5) and also the most severe substrate inhibition among all investigated substrates. The results suggest (–)-epicatechin as a physiological substrate of *Dl*PPO1. This work provides insights into the biochemical and catalytic properties of longan PPO that will be useful for future post-harvesting interventions for effective management of costly fruit browning reactions.

## Materials and methods

Unless specified differently, all used chemicals have been purchased from Sigma-Aldrich (Vienna, Austria) or Carl-Roth (Karlsruhe, Germany) and were at least of analytical grade. Enzymes for the manipulation of nucleic acids were from New England Biolabs (NEB; Frankfurt am Main, Germany) or Thermo Fisher Scientific (Waltham, MA, USA).

### Plant material, cloning, and sequencing of *Dl*PPO1

Longan leaves were collected in Lamphun (18.481174° N, 99.176014° E), located in the northern part of Thailand, immediately transported to the laboratory and stored at − 80 °C. Permission to collect longan leaves for research purposes was obtained from the local proprietor. Healthy leaves were ground under liquid nitrogen and the total RNA was isolated using the Rapid CTAB method according to Gambino et al.^96^ with an extraction buffer containing 2%(m/v) cetyltrimethylammonium bromide, 2.5%(m/v) polyvinylpyrrolidone, 2 M NaCl, 100 mM Tris–HCl (pH 8.0), 25 mM ethylenediaminetetraacetic acid (EDTA, set to pH 8.0 with NaOH) and 2%(v/v) β-mercaptoethanol. Subsequently, cDNA was synthesized using a poly-T primer (5’-T_25_VN-3’) and Moloney Murine Leukemia Virus reverse transcriptase. Specific primers for the longan PPO (prepro-*Dl*PPO1 and pro-*Dl*PPO1) gene were designed (Table S2). The primers were used to amplify the PPO genes from the cDNA template using the Q5 High-Fidelity DNA polymerase (NEB). PCR products were cloned into the pGEX-6P-SG^69^ expression vector by the following procedure: The pGEX-6P-SG vector (1 fmol) was incubated with 5 fmol of the purified prepro-*Dl*PPO1 or 5 fmol of pro-*Dl*PPO1 amplicon, 6 mM adenosine triphosphate (ATP), 4 units of Esp3I (Thermo Fisher Scientific), and 160 units of T4 DNA ligase (NEB) in a total volume of 6 μL 1 × CutSmart buffer (NEB) for 90 min at 30 °C, followed by enzyme inactivation at 65 °C for 20 min. The obtained plasmids were transformed into chemically competent SHuffle T7 Express cells (NEB). Transformants were selected on LB agar plates supplemented with 100 mg L^−1^ ampicillin by incubation at 37 °C overnight. Positive clones were detected with colony PCR and verified using Sanger-sequencing, carried out by the Microsynth GmbH (Vienna, Austria).

### Heterologous expression and purification of recombinant prepro-*Dl*PPO1 and pro-*Dl*PPO1

In the recombinant plasmids, the prepro-*Dl*PPO1 and pro-*Dl*PPO1 genes are N-terminally fused with the GST-tag of the pGEX-6P-SG vector. The human rhinovirus 3C protease (HRV3C) recognition sequence (LEVLFQ|GP) is located between the GST-tag and the target gene, enabling the controlled proteolytic dissociation of the tag from the protein of interest upon purification. The two fusion genes (GST-prepro-*Dl*PPO1 and GST-pro-*Dl*PPO1) were efficiently overexpressed using the synthetic *tac* promoter of the pGEX-6P-SG vector. The SHuffle T7 Express *E. coli* strain was grown in a modified 2xYT medium (1.6%(m/v) tryptone-peptone, 1%(m/v) yeast extract, 1%(m/v) NaCl, 0.5%(m/v) NH_4_Cl, 0.5%(v/v) glycerol, 2 mM MgCl_2_, 1 mM CaCl_2_; set to pH 7.5) in the presence of ampicillin (100 mg/L). Overnight cultures were grown at 37 °C. The expression batches were inoculated with the saturated overnight cultures and grown (starting OD_600_ of 0.05) at the same temperature for ~ 4 h until the OD_600_ reached a value of 0.6–0.8. To optimize the protein quantity and simultaneously to avoid inclusion body formation, the temperature was reduced to 17.5 °C for expression. The cultures were induced with 0.5 mM IPTG, and 2.0 mM CuSO_4_ were added. The expression cultures remained at 17.5 °C under shaking for 48 h. When the OD_600_ reached a value of 7–10, the cultures were collected by centrifugation at 6500 × *g* for 25 min at 4 °C.

Cell lysis was carried out implementing the freeze–thaw technique using liquid nitrogen. The pellets were resuspended in lysis buffer (50 mM Tris–HCl pH 7.5, 200 mM NaCl, 1 mM EDTA and 50 mM sucrose). Lysozyme (0.5 g/L) and protease inhibitors (1 mM phenylmethylsulfonyl fluoride and 1 mM benzamidine) were added to lyse the bacterial cell walls and to prevent protein degradation by endogenous proteases, respectively. The suspensions were shaken and incubated on ice for 45 min. The mixtures were then subjected to five cycles of freezing in liquid nitrogen, alternating with thawing in a 25 °C water bath. After the 5th round of the freeze–thaw procedure, 2 mM MgCl_2_ and 0.02 g/L DNaseI were added to remove the viscosity of the solutions caused by the DNA that was released from the bacteria cells and to improve protein extraction. The lysates were subsequently centrifuged at 7500 × *g* for 1 h at 4 °C.

Chromatographic purifications were carried out using an ÄKTA Purifier (GE Healthcare) placed in a refrigerator at 4 °C. The filtrated lysates were placed in a 50 mL injection loop and applied onto a prepacked 5 mL GSTrap FF column using a solution of 50 mM Tris–HCl, 250 mM NaCl pH 7.5 as the binding buffer. The target GST-fusion proteins were trapped onto the column, whereas the unbound proteins were flushed out by the binding buffer applied at a flowrate of 1 mL/min and chaperone washing buffer (50 mM Tris–HCl, 250 mM NaCl, 10 mM MgCl_2_ and 5 mM Na-ATP set to pH 8; applied for 15 column volumes) to remove binding chaperones from the target fusion proteins. Eventually, the desired proteins were eluted with 50 mM Tris–HCl, 250 mM NaCl and 15 mM reduced glutathione set to pH 7.5. The GST-fusion protein fractions were pooled and concentrated using a Vivaspin ultracentrifugation device with a 30 kDa molecular weight cut-off (Sartorius; Göttingen, Germany). To remove glutathione, the buffer was then exchanged to 50 mM Tris–HCl pH 7.5 and 250 mM NaCl. Subsequently, the samples were mixed with an in-house^3^ prepared GST-HRV3C protease at a mass ratio of 1:50 (protease: GST-fusion protein). The proteolysis was carried out over 48 h at 4 °C. Afterwards, the cleaved proteins were again applied onto a 5 mL GSTrap FF column. At this time, the GST-protein and the GST-tagged protease were trapped in the column while the PPOs were able to flow through the column and were immediately eluted. The protein fractions of prepro-*Dl*PPO1 and pro-*Dl*PPO1 were collected, concentrated and stored in 100 mM MES and 200 mM NaCl pH 6.5 with 30%(v/v) glycerol at -80 °C. The protein concentrations were determined using the Beer-Lambert law^97^ based on the absorption at 280 nm and the extinction coefficient provided by ExPASy ProtParam^98^. The predicted molecular mass of the expressed proteins were estimated from the atomic weight and standard isotopic composition of the constituent elements^74^. The copper contents of the prepro-*Dl*PPO1 and pro-*Dl*PPO1 enzymes were assessed photometrically after acidification with acetic acid and reducing Cu(II) to Cu(I) with ascorbate, which allowed for the chelation of Cu(I) by 2,2′-biquinoline according to the method published by Hanna et al.^73^.

### Molecular mass determination by ESI-LTQ-Orbitrap-Velos

Mass determination of prepro-*Dl*PPO1 and pro-*Dl*PPO1were performed by an ESI-LTQ-Orbitrap Velos (Thermo Fisher Scientific Bremen, Germany) mass spectrometer with a mass range of 200–4000 m/z and a mass accuracy close to 3 ppm with external calibration. Prior to MS 100 µg of the protein solutions were exchanged into 5 mM sodium acetate (pH 7.0) buffer. The protein solutions were diluted 100-fold in a mixture of 80%(v/v) acetonitrile and 0.1%(v/v) formic acid immediately before being applied to the mass spectrometer.

### Gel electrophoresis

Denaturing SDS-PAGE was performed as described by Laemmli et al*.*^99^ in a mini gel apparatus (Mini-PROTEAN Tetra Cell, Bio-Rad). The prepro-*Dl*PPO1 and pro-*Dl*PPO1 were diluted in gel loading buffer that contains 30 g/L SDS, 6% (v/v) glycerol, 75 mM Tris, 2.5%(v/v) 2-mercaptoethanol and 50 mg/L bromophenol blue set to pH 6.8. The samples were heated at 99 °C (Thermomixer comfort, Eppendorf) for 5 mins to aid in the denaturation. The samples were then applied on the 5% stacking and 11% resolving polyacrylamide gels along with a molecular weight standard (Precision Plus Protein Standard Dual Color, Bio-Rad) to provide information on the size and the purity of the proteins. The electrophoresis was run at 120 V. Gels were stained in dye solution (200 mg/L Coomassie brilliant blue G-250, 50 g/L Al_2_(SO_4_)_3_^**.**^16 H_2_O, 10%(v/v) ethanol and 20 g/L H_3_PO_4_) and were subsequently destained with 10%(v/v) ethanol and 20 g/L *ortho*-phosphoric acid. Photographs of the gels were taken using the BioRad Gel Doc XR Imaging System.

### Thermal shift assay of prepro-*Dl*PPO1 and pro-*Dl*PPO1

The thermal shift assay was conducted to measure the melting points of the two isoenzymes in the storage buffer (50 mM MES pH 6.5 and 200 mM NaCl) in order to determine the stability of the two states of the enzyme. The assay was performed thrice using PCR tubes (Axygen, INC Corning) in a real-time PCR instrument (mastercycler ep-realplex, Eppendorf). The reaction solutions contained 10 µM enzyme and 4 × SYPRO Orange (Sigma-Aldrich) in the storage buffer. The samples were gradually heated with an increment of one K per minute from 4 °C to 94 °C in the PCR machine, while the change in fluorescence intensity was monitored at 560 nm following excitation at 470 nm. The resulting fluorescence intensities of the solutions containing each enzyme were then plotted against the temperature for stability analysis.

### Enzyme activity assays

The *Dl*PPO1 activities were spectrophotometrically determined by monitoring the increase in absorbance at 480 nm upon the conversion of tyramine by the enzyme. The standard measurements were performed using 0.5 µg of the enzyme and 8 mM of tyramine in a total reaction volume of 200 µL with the determined optimal SDS concentration (0.25 mM) to activate prepro-*Dl*PPO1 or pro-*Dl*PPO. In the absence of SDS no utilizable absorption-time curves were obtained for the tested substrates (Figure S12). Absorption curves and spectra were recorded at 25 °C in a 96 well microplate applying a TECAN infinite M200 (Tecan). The activities were determined from the slope of the initial linear part of the experimental curves (absorbance vs. time) and expressed as U/min. One unit of enzymatic activity (U) was defined as the amount of enzyme that catalyzed the formation of 1 μmol of quinones per minute (1 U = 1 μmol/min). All assays were performed in triplicate. Optimizations of the SDS concentration and pH of the reaction buffer towards the pro-*Dl*PPO1 activity are as follows (vide infra).

### Activation of pro-*Dl*PPO1

#### Effect of SDS on pro-*Dl*PPO1 activity

The measurements (200 µL) were performed in the presence of various SDS concentrations (0.00, 0.05, 0.10, 0.25, 0.50, 0.75, 1.00, 1.25 and 1.50 mM) using 0.5 µg pro-*Dl*PPO1 and 8 mM tyramine in 50 mM MES at pH 7.0. The optimal SDS concentration was subsequently used in all other following studies.

#### Effect of pH on pro-*Dl*PPO1 activity

A series of MES and Tris buffers over a pH range of 5.5–8.5 were used as the reaction buffer to determine the most favorable pH that provides the best enzyme activity for further kinetics studies. Each reaction (200 µL) was carried out with 50 mM buffer concentration using 0.5 µg pro-*Dl*PPO1, 8 mM tyramine and the optimal SDS concentration obtained from the previous experiment.

#### Substrate specificity and kinetic efficiency determination

Substrate specificity of prepro-*Dl*PPO1 and pro-*Dl*PPO1 was probed using various phenolic substrates. The substrates that resulted in decent activities were subsequently selected to determine kinetic parameters. Activities were evaluated spectrophotometrically on two standard monophenolic substrates (tyramine, *l*- and *d-*tyrosine), two standard diphenolic compounds (dopamine and *l*-DOPA) and four natural phenolic substrates ((–)-epicatechin, 4-methylcatechol, pyrogallol and caffeic acid). The absorption curves of the colored products were monitored on a TECAN infinite M200 for different molarities of the respective substrate. The reaction mixture (total volume of 200 µL) contained variable amounts of the enzyme (see Table S4, stored in 100 mM MES and 200 mM NaCl at pH 6.5) and 0.25 mM SDS in the reaction solution of 50 mM MES buffer at pH 7. The molar absorption coefficients (ɛ_λmax_) of the formed chromophores are reported in Table S4 and Figure S19. To perform the kinetic experiments, about 7–8 substrate concentrations around a preliminary K_M_ value were chosen if the limited substrate solubility allowed to do so (Figures S13-S16). K_M_ and v_max_ values were calculated by nonlinear regression using the Levenberg–Marquardt algorithm^100^ that was supplied with initial values for the model parameters derived from applying the Hanes-Woolf linearization^101^ of the Michaelis–Menten equation (Equation S1). The maximal turnover rate (k_cat_, Equation S2) was evaluated by dividing the total number of substrate molecules that were converted per min by the total number of enzyme molecules present in the reaction volume. For combinations of enzyme and substrate showing substrate inhibition the Michaelis–Menten equation was extended to include inhibition by the substrate (Equation S3).

#### Formation of the *oxy*-adduct

The prepro-*Dl*PPO1 and pro-*Dl*PPO1 were spectrophotometrically investigated using H_2_O_2_ that induces the formation of the characteristic *oxy*-state of type-III copper enzymes^102^. Addition of H_2_O_2_ to the prepro-*Dl*PPO1 or pro-*Dl*PPO1 leads to the formation of a new absorption band around ~ 345 nm which is characteristic for the peroxide-induced *oxy*-state of the dicopper center^87^. In these experiments 255 µg of pro-*Dl*PPO1 (ε_280_ = 74,300 M^−1^ cm^−1^) or 300 µg of prepro-*Dl*PPO1 (ε_280_ = 74,425 M^−1^ cm^−1^) were mixed into 200 µL of the storage buffer (100 mM MES and 200 mM NaCl pH 6.5). Several equivalents of H_2_O_2_ were added to the solution until saturation of the characteristic peak at ~ 345 nm was reached. After each addition of another equivalent of H_2_O_2_ to the investigated enzyme, enough time (but at least 5 mins) was allowed until absorption at the characteristic wavelength of 345 nm was stable and only then the next equivalent of H_2_O_2_ was added. The measurement finished when the characteristic peak (~ 345 nm) did not increase any further with additional H_2_O_2_.

#### Homology modelling of pro-*Dl*PPO1

The amino acid sequence of the pro-*Dl*PPO1 (Ala1-Asp503) was submitted to the SWISS-MODEL^103,104^ server. At the beginning, the pipeline searched for an appropriate template of the investigated sequence (OU702517) based on BLAST^105^ and HHblits^106^. The search resulted in *Md*PPO1^66^, *Malus domestica* TYR 1 (PDB: 6ELS)^35,36^, as the hit which exhibited the highest coverage value (0.86, with 69.80% sequence identity). The final homology model of the pro-*Dl*PPO1 was then created using the 6ELS structure as the template and visualization was done using the PyMol Molecular graphic system (Schrödinger, LLC)^107^.

#### Molecular docking

Molecular docking was done using the AutoDock Vina software^94^. Docking studies were used to identify the binding poses of the investigated natural substrates (–)-epicatechin, 4-methylcatechol, caffeic acid, pyrogallol and the standard substrates dopamine, *l*-DOPA, tyramine, *l*-tyrosine and *d*-tyrosine in the dicopper active center of pro-*Dl*PPO1. The molecular model of the pro-*Dl*PPO1 was prepared for molecular docking as described before for *Md*PPO1^66^. Specifically, from the putative structure of the pro-*Dl*PPO1 the C-terminal domain (Pro336-Asp503) was removed and only the active domain (Ala1-Val335) was used for the docking studies. Moreover, the gatekeeper residue (Phe260) was defined as a flexible residue, as its flexibility has been verified for plant PPOs^17^. The structure of all the substrates were downloaded from the PubChem server^108^, then translated to the 3D pdb format and converted into pdbqt files using AutoDockTools (ADT, v. 1.5.6)^109^. For all substrates the highest possible number of active torsions was chosen. Polar hydrogens were assigned to the structures by ADT and were also saved in the substrate pdbqt files. In all the experiments, the search grid was centered in the middle of the two active site copper ions and spread over a cube (12 × 12 × 12 Å^3^, grid point spacing: 1 Å) with edges parallel to the three coordinate axes of the target *Dl*PPO1 model. Then AutoDock/Vina was used for docking by supplying the investigated enzymes and inhibitors along with the grid box properties in the configuration file. For all the measurements the energy range was set to 5, while the exhaustiveness was set to 50. Upon docking, the binding poses were evaluated by superimposing the co-crystallized substrate *l*-tyrosine from the *Bm*TYR crystal structure (PDB: 4P6R)^1^. Poses that grossly deviated from the binding pose of *l*-tyrosine in the *Bm*TYR structure (*i.e*. substrate poses with the phenolic ring not interacting with the dicopper center) were characterized as ‘unreasonable’ poses. Reasonable docking configurations (the phenolic ring linked to the copper ions similar to *Bm*TYR (PDB: 4P6R)^1^) were plotted as a 3D image using the visualization software PyMol Molecular graphic system (Schrödinger, LLC)^107^.

#### Generation of interaction plots

After the docking study, reasonable binding poses of interest were saved as pdb files and further visualized using the LigPlot + software^110,111^ to evaluate the interactions between the ligand (–)-epicatechin and the interacting amino acid residues around the active center. Runtime parameters were defined as suggested by the software. Specifically, hydrogen-bonds were allowed up to a maximal range of 2.70 Å between a hydrogen and a hydrogen acceptor and also up to a maximal range of 3.35 Å between a hydrogen donor and a hydrogen acceptor. Non-bonded contact parameters were defined as maximal distances of 2.90 Å between the hydrophobic carbons of the ligand and the protein and of 3.90 Å between sulfur atoms of the ligand and the protein.

### Ethical approval

All plant experiments described in this study complied with the relevant institutional, national, and international guidelines and legislation.

## Supplementary Information

Supplementary Information.

## Data Availability

The sequences generated and analyzed during the presented study are available from the European Nucleotide Archive (ENA) repository under the accessions OU702517 (partial pro-*Dl*PPO1 gene) and OU702518 (prepro-*Dl*PPO1 gene).
