# Supplementary material for: Biochemical characterization of Dimocarpus longan polyphenol oxidase provides insights into its catalytic efficiency
Source: Sci Rep. 2022 Nov 25;12:20322. doi: 10.1038/s41598-022-20616-7 (PMC9700842; doi:10.1038/s41598-022-20616-7)
Supplement: Supplementary file 1 — Supplementary Information. [file 41598_2022_20616_MOESM1_ESM.docx]

**Supplementary Information**

**Biochemical Characterization of *Dimocarpus longan* Polyphenol Oxidase Provides Insights into its Catalytic Efficiency**

Leela Ruckthong^a,b^, Matthias Pretzler^a^, Ioannis Kampatsikas^a^ and Annette Rompel^a,*^

^a^ Universität Wien, Fakultät für Chemie, Institut für Biophysikalische Chemie, Josef-Holaubek-Platz 2, 1090 Wien, Austria; www.bpc.univie.ac.at

^b^ King Mongkut’s University of Technology Thonburi (KMUTT), Faculty of Science, Department of Chemistry, Thung Kru, Bangkok 10140, Thailand

*Correspondence to: [annette.rompel@univie.ac.at](mailto:annette.rompel@univie.ac.at)

**26 Pages**

**4 Tables**

**19 Figures**

**3 Equations**

| **Contents** | **Page** |
| --- | --- |
| **1. List of Abbreviations** | **3** |
| **2. Tables** | **4** |
| Table S1 INSDC Database entries for the sequences of the recombinant longan enzyme. | 4 |
| Table S2 Primers used to amplify the expressed genes and their GC content. | 4 |
| Table S3 Binding energies of the investigated substrates docked into the dicopper active center of *Dl*PPO1. | 5 |
| Table S4 Amounts of *Dl*PPO1 (µg), wavelength (nm) and molar extinction coefficients (M^-1^ cm^-1^) used for kinetic measurements of phenolic compounds. | 5 |
| **3. Figures** | **6** |
| Figure S1 A) Schematic representation of the recombinant prepro-*Dl*PPO1, pro-*Dl*PPO1 and the active domain of *Dl*PPO1.  B) Structural representation of the pro-*Dl*PPO1 active site center modelled based on the wild-type *Md*PPO1 (PDB: 6ELS). | 6 |
| Figure S2 Structural formulae of kinetically tested natural substrates of *D. longan* PPO. | 7 |
| Figure S3 Structural formulae of kinetically tested standard substrates of *D. longan* PPO. | 8 |
| Figure S4 Gel-purified PCR products of Q5 High-Fidelity DNA-polymerase on *D. longan* cDNA. | 9 |
| Figure S5 Sequence alignment between the recombinant longan PPOs (pro-*Dl*PPO1 and prepro-*Dl*PPO1) and the previously published *Dl*PPO1 sequence. | 10 |
| Figure S6 Untruncated SDS-PAGE gel showing the effect of expression temperature on the amount of soluble *Dl*PPO1 proteins obtained from the bacterial cell culture. | 11 |
| Figure S7 Typical chromatograms of GST-affinity purifications of pro-*Dl*PPO1 and prepro-*Dl*PPO1. | 12 |
| Figure S8 Full-length SDS-PAGE gels of pro-*Dl*PPO1 (left) and prepro-*Dl*PPO1 (right) at different stages of purification. | 13 |
| Figure S9 Fluorescence curves of the thermal shift assay of pro-*Dl*PPO1 and prepro-*Dl*PPO1. | 14 |
| Figure S10 Effect of SDS concentration on prepro-*Dl*PPO1 and pro-*Dl*PPO1 activities. | 15 |
| Figure S11 pH-dependence of the reaction rate of SDS-activated pro-*Dl*PPO1 in MES and TRIS buffers. | 16 |
| Figure S12 Activity of pro-*Dl*PPO1 and prepro-*Dl*PPO1 without activator. | 17 |
| Figure S13 Michaelis-Menten diagrams of the enzymatic reactions of pro-*Dl*PPO1 applying standard phenolic substrates. | 18 |
| Figure S14 Michaelis-Menten diagrams of the enzymatic reactions of prepro-*Dl*PPO1 applying standard phenolic substrates. | 19 |
| Figure S15 Michaelis-Menten diagrams of the pro-*Dl*PPO1 enzymatic reactions applying natural phenolic substrates. | 20 |
| Figure S16 Michaelis-Menten diagrams of the prepro-*Dl*PPO1 enzymatic reactions applying natural phenolic substrates. | 21 |
| Figure S17 Structural comparison of the interactions between the *Dl*PPO1 active site and the natural substrates obtained from the docking studies. | 22 |
| Figure S18 Structural comparison of the interactions between the *Dl*PPO1 active site and the standard substrates obtained from docking studies. | 23 |
| Figure S19 Absorbances obtained by oxidation of phenolic substrates to determine the molar extinction coefficients using linear curve fitting. | 24 |
| **4. Equations** | **25** |
| Equation S1 Michaelis-Menten kinetics. | 25 |
| Equation S2 Maximal turnover rate *k_cat_*. | 25 |
| Equation S3 Michaelis-Menten kinetics with substrate inhibition. | 25 |
| **5. References** | **25** |

**1. List of Abbreviations**

cDNA complementary Deoxyribonucleic Acid

*Dl*PPO1 *Dimocarpus longan* Polyphenol Oxidase 1

DNA Deoxyribonucleic Acid

GC Guanine or Cytosine

GST *Schistosoma japonicum* Glutathione-S-transferase

*Md*PPO1 *Malus domestica* Polyphenol Oxidase 1

MES 2-(N-morpholino)ethanesulfonic acid

*l*-DOPA Levodopa (l-3,4-dihydroxyphenylalanine)

PCR Polymerase Chain Reaction

PPO Polyphenol Oxidase

SDS-PAGE Sodium Dodecyl Sulfate Polyacrylamide Gel Electrophoresis

TRIS Tris(hydroxymethyl)aminomethane

### 1. Tables

### Table S1. INSDC Database^1^ entries for the sequences of the recombinant longan enzyme.

| **PPO** | **Accession number** | **Size**  **[bp]** | **ORF**  **[AA]** | **MW (cal.)***  **[Da]** |
| --- | --- | --- | --- | --- |
| pro-*Dl*PPO1 | OU702517 | 1512 | 503 | 56844.05* |
| prepro-*Dl*PPO1 | OU702518 | 1800 | 599 | 67284.68* |

* The predicted molecular mass (MW) values of the expressed proteins were estimated with the inclusion of the vector-derived GlyProMet residues that remain at the N-terminus of the protein after proteolysis with the HRV3C protease, the presence of one thioether bridge (-2H) and two disulfide bridges (-4H). The prediction was calculated from the respective protein’s sum formula, the atomic weight and isotopic composition of the constituent elements.^2^

### Table S2. Primers used to amplify the expressed genes and their GC content.

| **PPO** | **Primers** | **%GC** |
| --- | --- | --- |
| pro-*Dl*PPO1 | Forward:  5’- AGCTcgtctcCAATGGCTCCAGTTTCGGCCC -3’  Reverse:  5’- AGCTcgtctcATCCCTCAATCTTGGGCAAAATCAATCTTAATACC -3’ | 48  52 |
| prepro-*Dl*PPO1 | Forward:  5’- AGCTcgtctcCAATGATGGCTTCTTTCTCTCCAACATC -3’  Reverse:  5’- AGCTcgtctcATCCCTCAATCTTGGGCAAAATCAATCTTAATACC -3’ | 64  52 |

**Table S3: Binding energies of the investigated substrates docked into the dicopper active center of *Dl*PPO1.**

| **Substrate** | **ΔG [kcal/mol]** |
| --- | --- |
| ***Natural substrates**** |  |
| 4-methylcatechol | -2.7 |
| caffeic acid | -2.1 |
| pyrogallol | -3.9 |
| (–)-epicatechin | -7.1 |
| ***Standard substrates***** |  |
| dopamine | -1.8 |
| *l*-DOPA | -2.3 |
| tyramine | -2.8 |
| *l*-Tyrosine | -2.4 |
| *d*-Tyrosine | -2.2 |

The structural formulae of the phenolic substrates are shown in **Figure S2** (natural substrates) and **Figure S3** (standard substrates).

* “Natural substrates” refers to phenolic compounds that have been observed in longan fruit.^3-5^

** “Standard substrates” refers to common phenolic compounds that are usually used to characterize PPOs.^6-10^

**Table S4. Amounts of *Dl*PPO1 (µg), wavelength (nm) and molar extinction coefficients (M^-1^ cm^-1^) used for kinetic measurements of phenolic compounds.**

| **State of enzyme** | **substrate** | ***Dl*PPO1 (µg)** | **λ_max_ (nm)** | **ε_max_ (M^-1^ cm^-1^)** |
| --- | --- | --- | --- | --- |
| pro-*Dl*PPO1 or  prepro-*Dl*PPO1 | tyramine | 0.50 | 480^a^ | 3300^a^ |
|  | *l*-tyrosine | 30.0 | 475^a^ | 3600^a^ |
|  | *d*-tyrosine | 30.0 | 475^a^ | 3600^a^ |
|  | *l*-DOPA | 0.50 | 475^a^ | 3600^a^ |
|  | dopamine | 0.50 | 480^a^ | 3300^a^ |
|  | (-)-epicatechin | 0.25 | 440^b^ | 4896^b^ |
|  | 4-methylcatechol | 0.50 | 373^b^ | 966^b^ |
|  | caffeic acid | 5.00 | 495^a^ | 2062^a^ |
|  | pyrogallol | 0.25 | 434^b^ | 1418^b^ |

^a^ Data reported in reference 11. ^b^ Data from **Figure S19**. The structural formulae of the phenolic substrates are shown in **Figure S2** (natural substrates) and **Figure S3** (standard substrates). “Natural substrates” refers to phenolic compounds that have been reported to be present in longan fruit.^3-5^

“Standard substrates” refers to common phenolic compounds that are usually used to characterize PPOs but are not reported as constituents of longan fruits.^6-10^

**2. Figures**

**
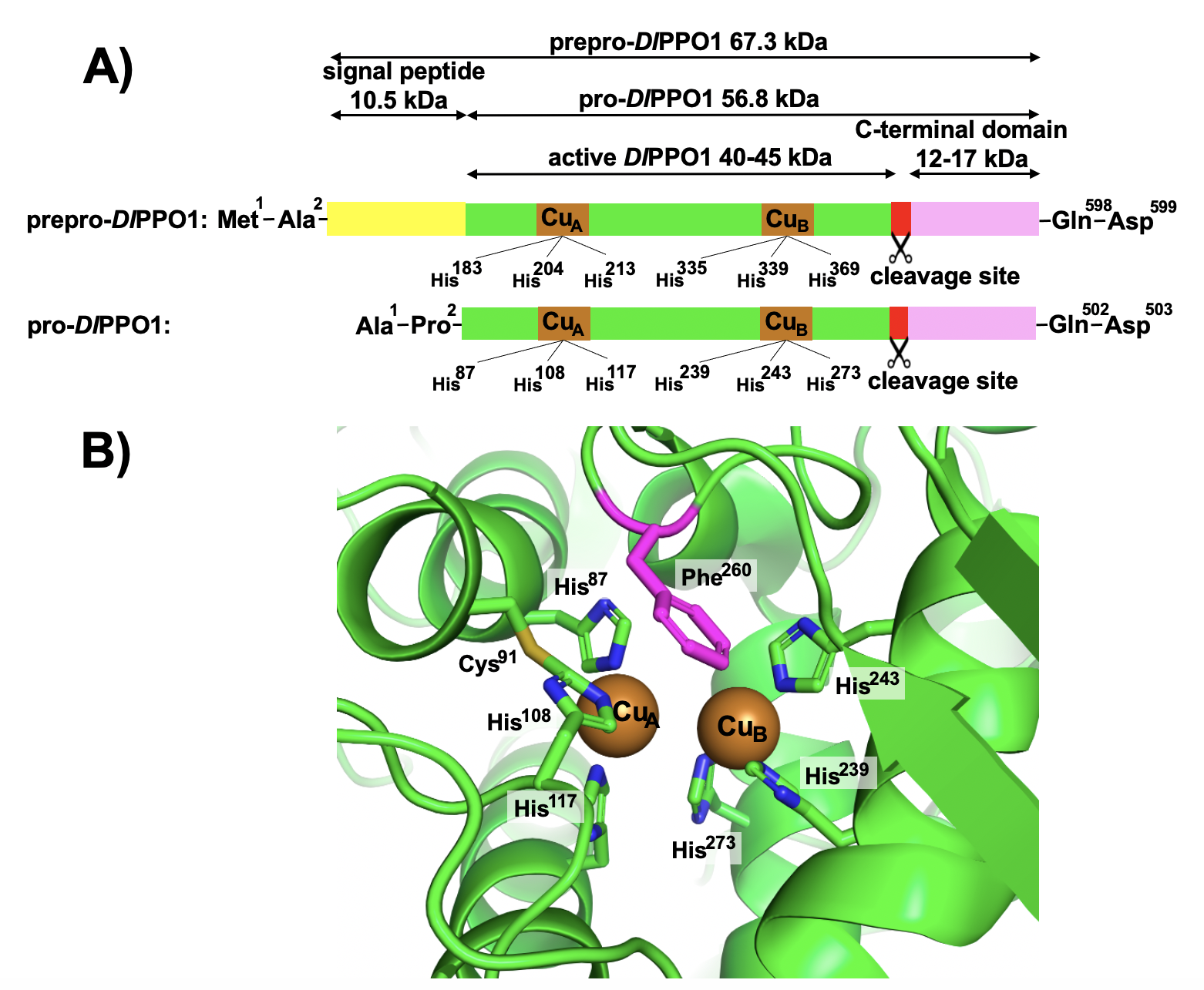
**

**Figure S1. A) Schematic representation of the recombinant prepro-*Dl*PPO1, pro-*Dl*PPO1 and the active domain of *Dl*PPO1.** The signal peptide of the enzyme is colored yellow, the active *Dl*PPO1 domain is colored green, the conserved active site regions are presented in brown, the putative proteolytic cleavage site is colored red, and the C-terminal domain is highlighted in pink. **B)** **Structural representation of the pro-*Dl*PPO1 active site center modelled based on the wild-type *Md*PPO1 (PDB: 6ELS)**^12^. The conserved histidine residues coordinating Cu_A_ (His^87^, His^108^ and His^117^) and Cu_B_ (His^239^, His^243^ and His^273^) are shown in green sticks (N atoms are shown in blue). The conserved gatekeeper phenylalanine residue (Phe^260^) is shown in pink. The thioether bridge between Cys^91^ and His^108^ (that is coordinated to Cu_A_) is shown in sticks with the S atom colored yellow. Both copper ions are represented as brown spheres.


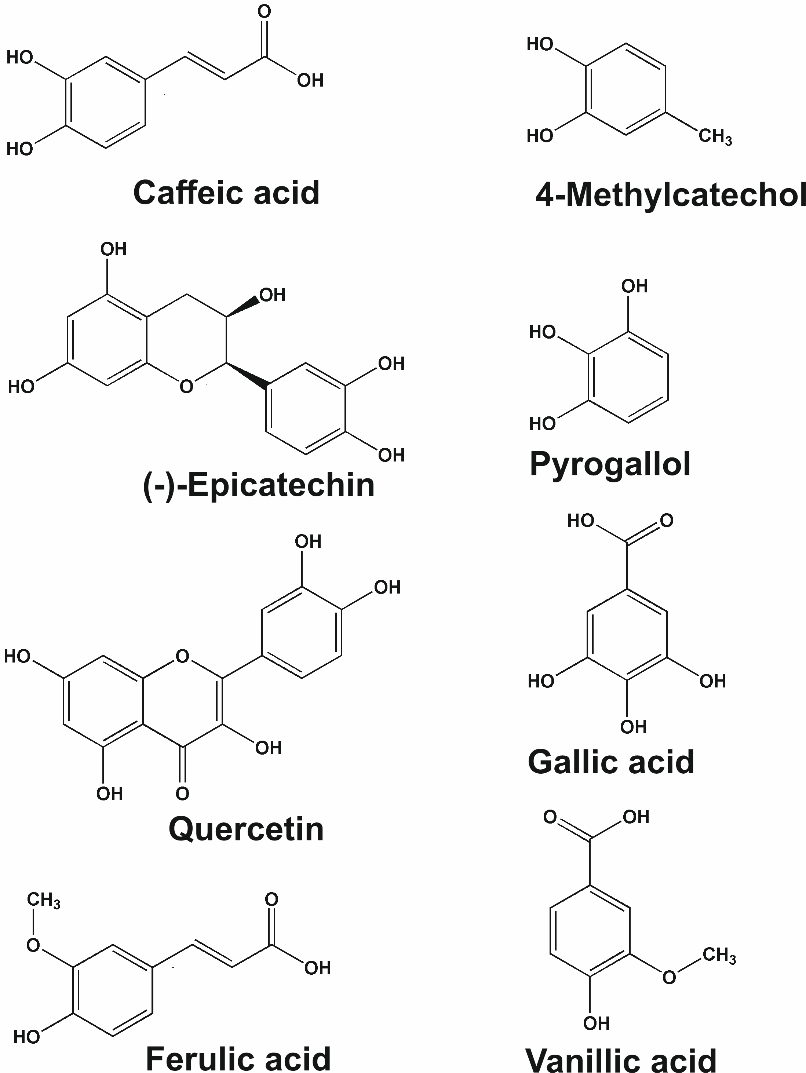


**Figure S2. Structural formulae of kinetically tested natural substrates of *D.* *longan* PPO*.*** “Natural substrates” refers to phenolic compounds that have been reported to be present in longan fruit.^3-5^


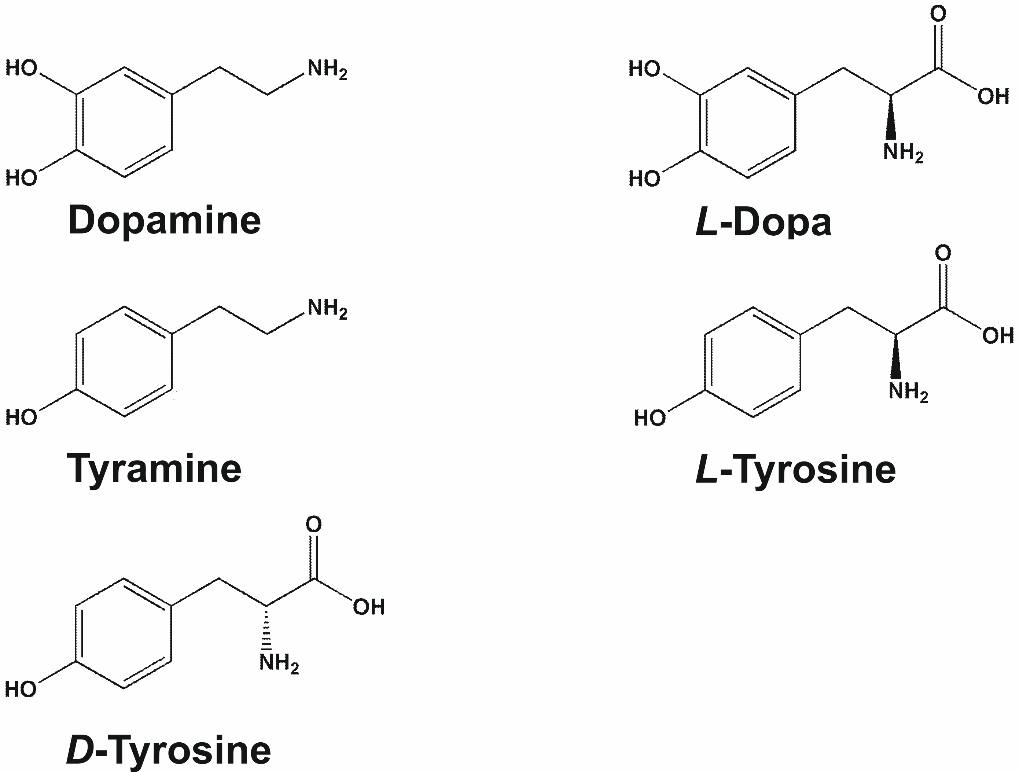

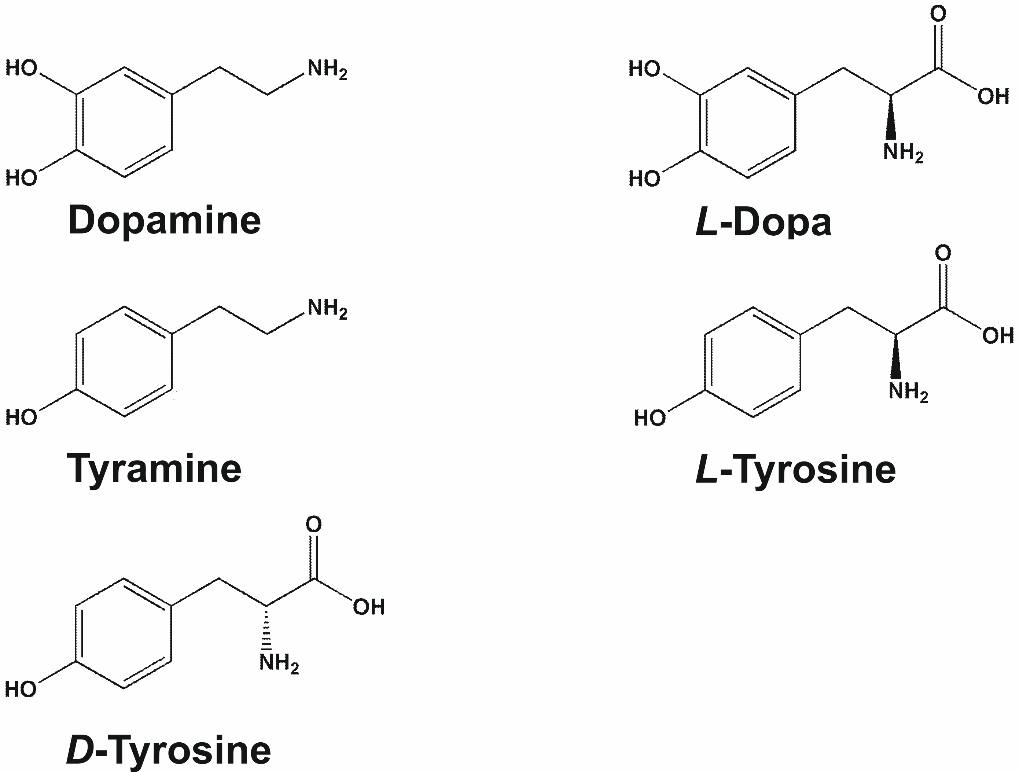


**Figure S3. Structural formulae of kinetically tested standard substrates** **of *D*. *longan* PPO.** “Standard substrates” refers to common phenolic compounds that are usually used to characterize PPOs but are not reported as constituents of longan fruits.^6-10^


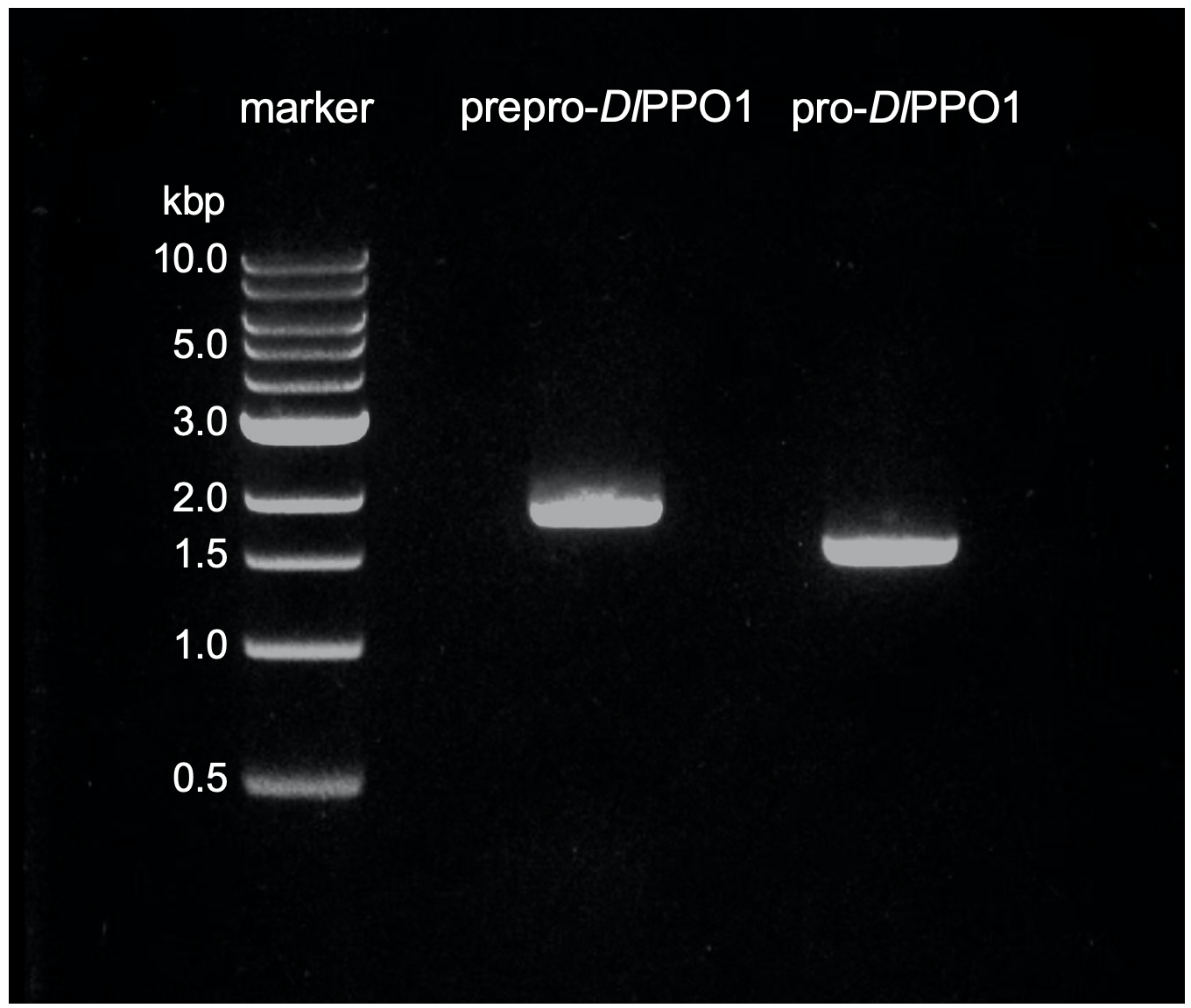


**Figure S4. Gel-purified PCR products of Q5 High-Fidelity DNA-polymerase on *D.* *longan* cDNA.** The two pairs of primers used in the study are listed in **Table S1**. The first lane represents the 1 kbp molecular size standard marker (NEB). The second and third lanes demonstrate the PCR products of prepro-*Dl*PPO1 (~1800 bp) and pro-*Dl*PPO1 (~1500 bp). The amplicons were run on a 1.0% (m/v) agarose gel. DNA amplicons were detected using the fluorescence of SYBR Safe DNA stain (Fisher Scientific, Vienna, Austria) with excitation at 302 nm.

**
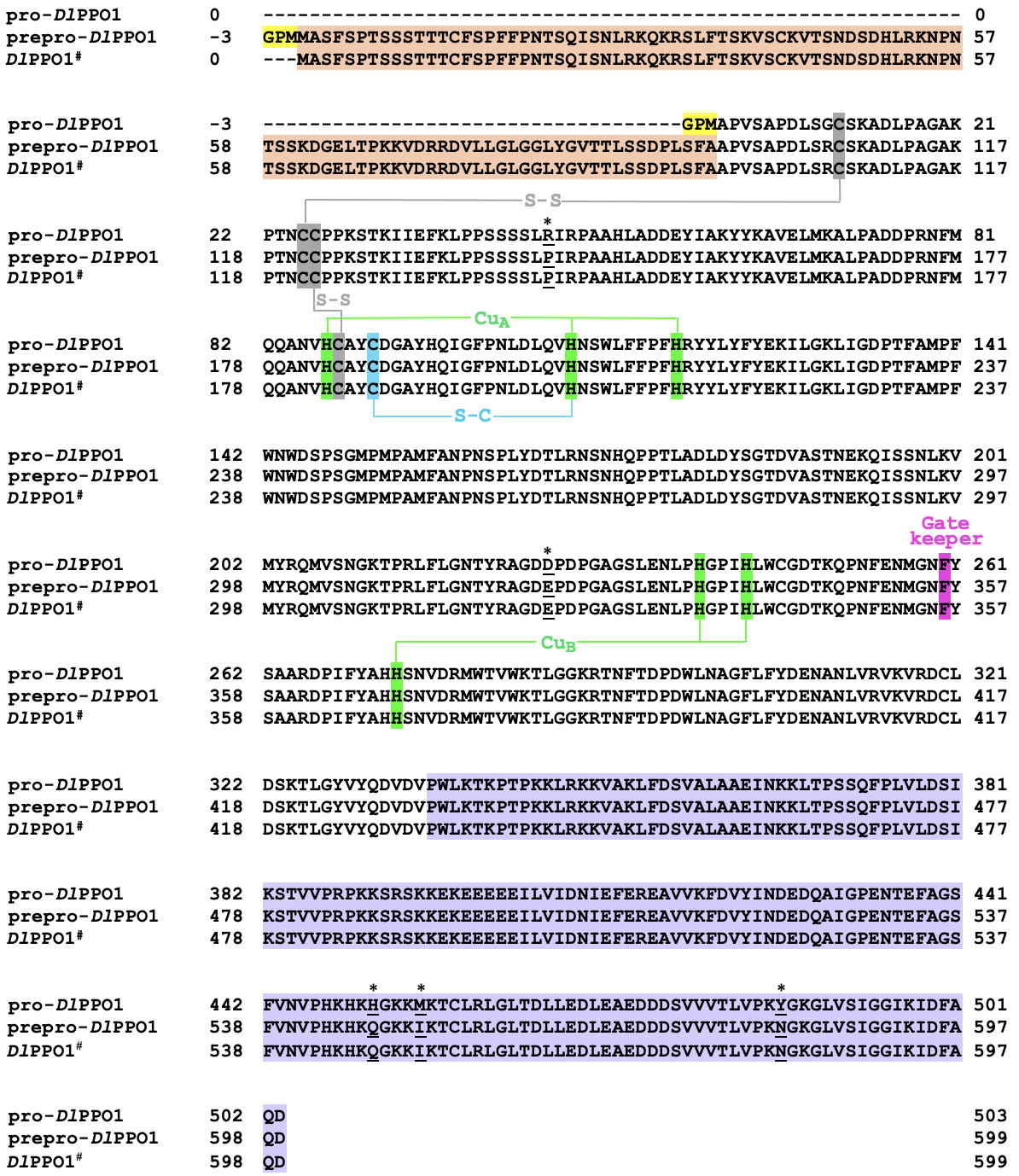
**

**Figure S5.** **Sequence alignment between the recombinant longan PPOs (pro-*Dl*PPO1 and prepro-*Dl*PPO1) and the previously published *Dl*PPO1 sequence^13^**. Highlighted are the conserved copper-coordinating histidines (green) of the dinuclear copper center, the conserved cysteines (grey and dark grey) that putatively form stabilizing disulfide bonds (S-S), the thioether bridge between a cysteine (blue) and the Cu_A_ coordinating histidine (S-C) as well as the conserved gatekeeper phenylalanine residue (pink). The orange region indicates the transit peptide sequence on the N-terminal side of the precursor prepro-*Dl*PPO1, while the C-terminal domain is colored purple. The yellow highlighted residues stem from the expression vector. Underlined and star-marked positions indicate amino acids that differ in the pro-*,* prepro-*Dl*PPO1 and the previously published *Dl*PPO1^13^. **^#^** indicates the sequence previously published (see reference 13).

**
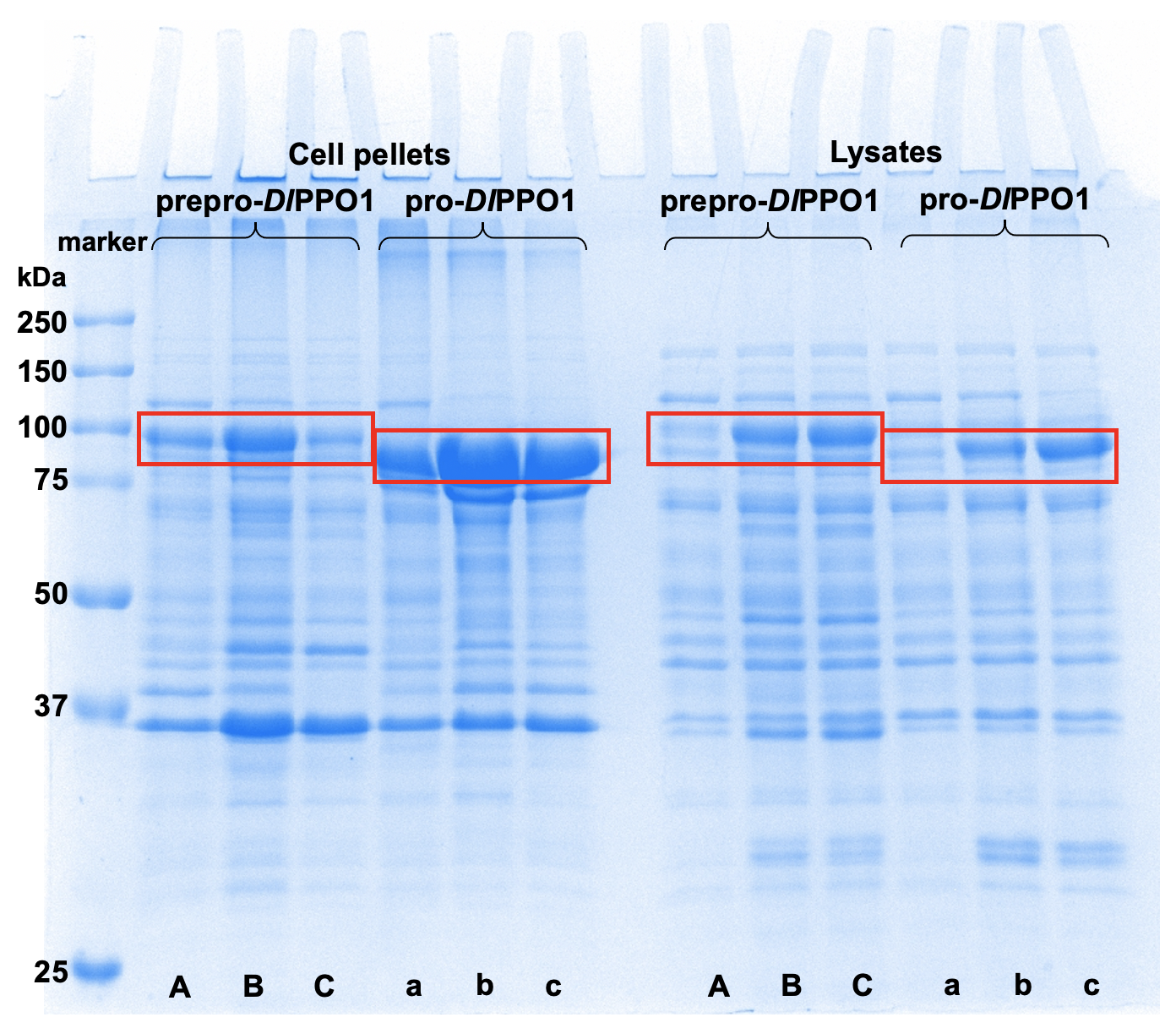
**

**Figure S6. Untruncated SDS-PAGE gel showing the effect of expression temperature on the amount of soluble** ***Dl*PPO1 proteins obtained from the bacterial cell culture.** The left panel represents the total insoluble proteins in the cell pellets and the right panel shows the total soluble proteins in cell lysates of the prepro-*Dl*PPO1 (**A**, **B**, **C**) and pro-*Dl*PPO1 (**a**, **b**, **c**) cell cultures. The temperatures at induction were 37 ^°^C (**A** and **a**), 28 ^°^C (**B** and b) and 17.5 ^°^C (**C** and **c**). The bands of interest are marked with red boxes. The lane indicated with “kDa” contains the molecular weight marker; the size of the standard bands is given in kDa.

**
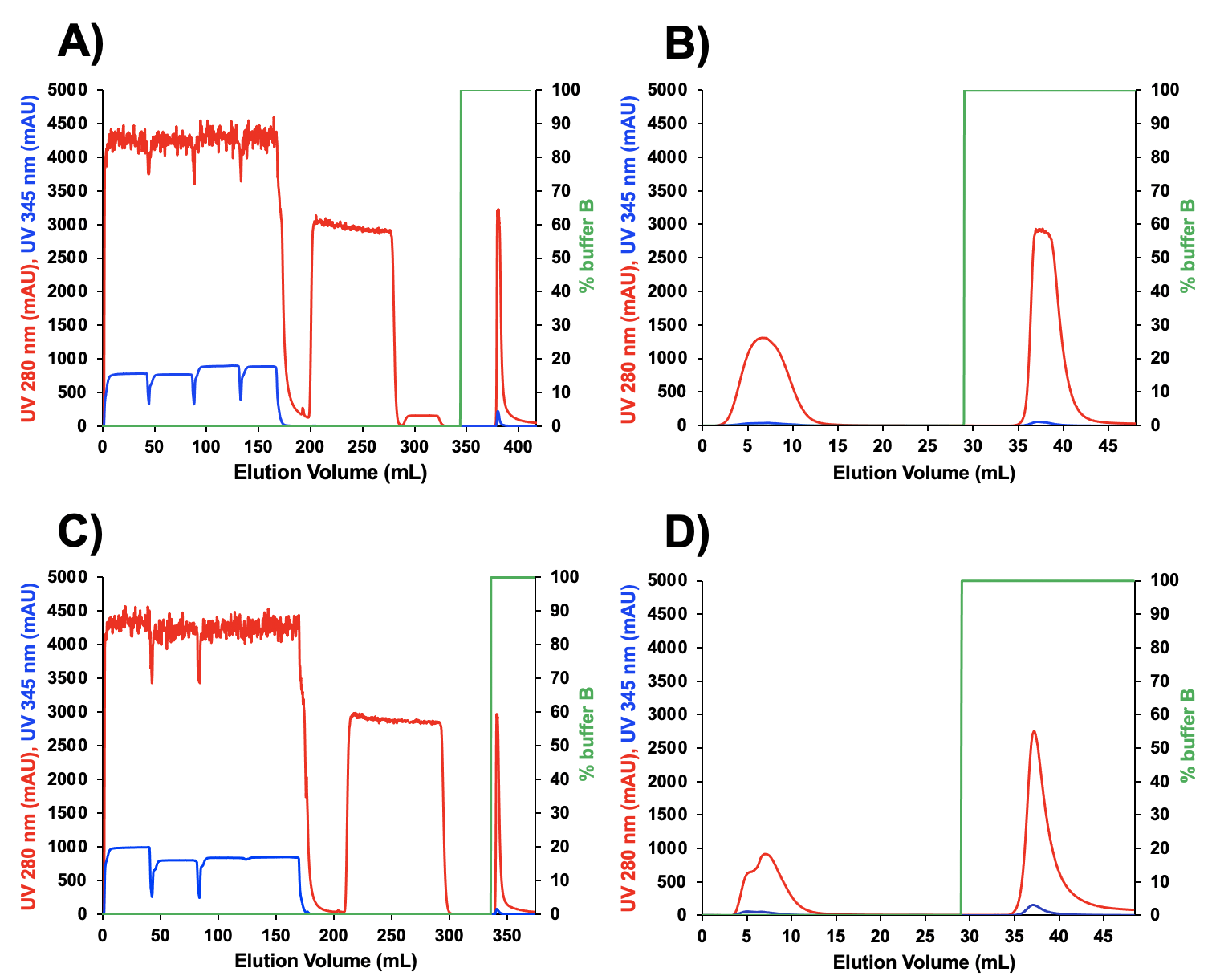
**

**Figure S7. Typical chromatograms of GST-affinity purifications of pro-*Dl*PPO1 and prepro-*Dl*PPO1.** Left panels represent the first purification step where the elution (last peak) of the GST-fusion with the pro-*Dl*PPO1 (**A**) and GST-fusion with the prepro-*Dl*PPO1 (**C**) took place. Fractions that were formerly eluted are other proteins that were produced by *E. coli*. The right panels demonstrate the second purification step of the pro-*Dl*PPO1 (**B**) and prepro-*Dl*PPO1 (**D**). The final protein product (first peak) eluted in the column flow-through, while the GST-tag and HRV3C protein (second peak) were eluted only in the presence of reduced glutathione. Legend: UV absorbance at 280 nm in mAU (red), UV absorbance at 345 nm in mAU (blue) and composition of the running buffer (% buffer B, green). Buffer B is the elution buffer that contains 15 mM reduced glutathione in 50 mM TRIS and 250 mM NaCl pH 7.5.

**
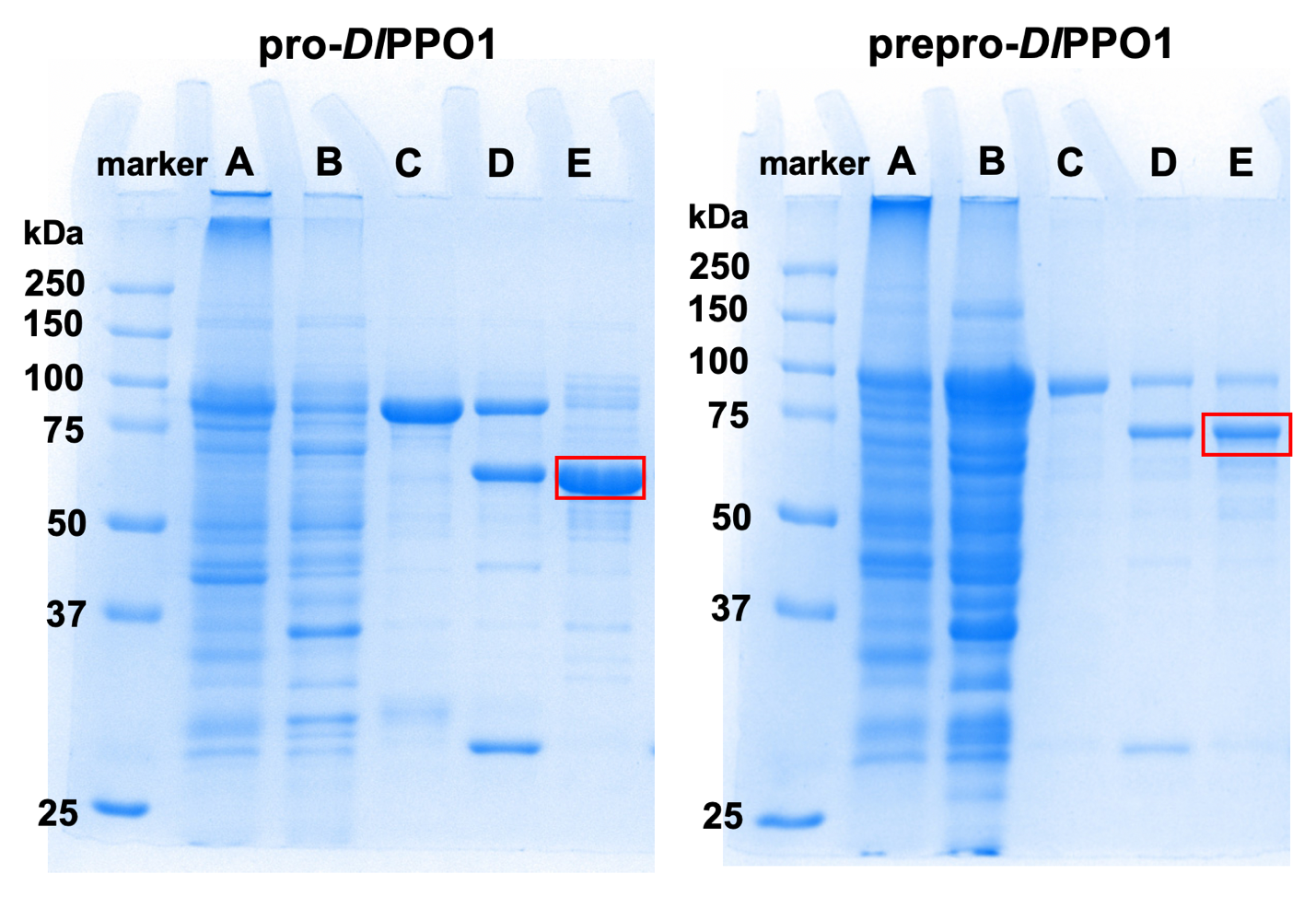
**

**Figure S8. Full-length SDS-PAGE gels of pro-*Dl*PPO1 (left) and prepro-*Dl*PPO1 (right) at different stages of purification.** **A**) insoluble fraction of the bacterial cell pellets, **B**) supernatant from cell lysates, demonstrating the total soluble proteins, **C**) GST-*Dl*PPO1 which eluted as a GST-fusion protein after glutathione affinity chromatography (first purification step on FPLC), **D**) fractions of proteins after the GST-*Dl*PPO1 was cut by the HRV3C protease^14^ and **E**) final *Dl*PPO1 product from the second round of glutathione affinity chromatography on FPLC (see Table S1). The first lane of each gel is the molecular weight marker.

###
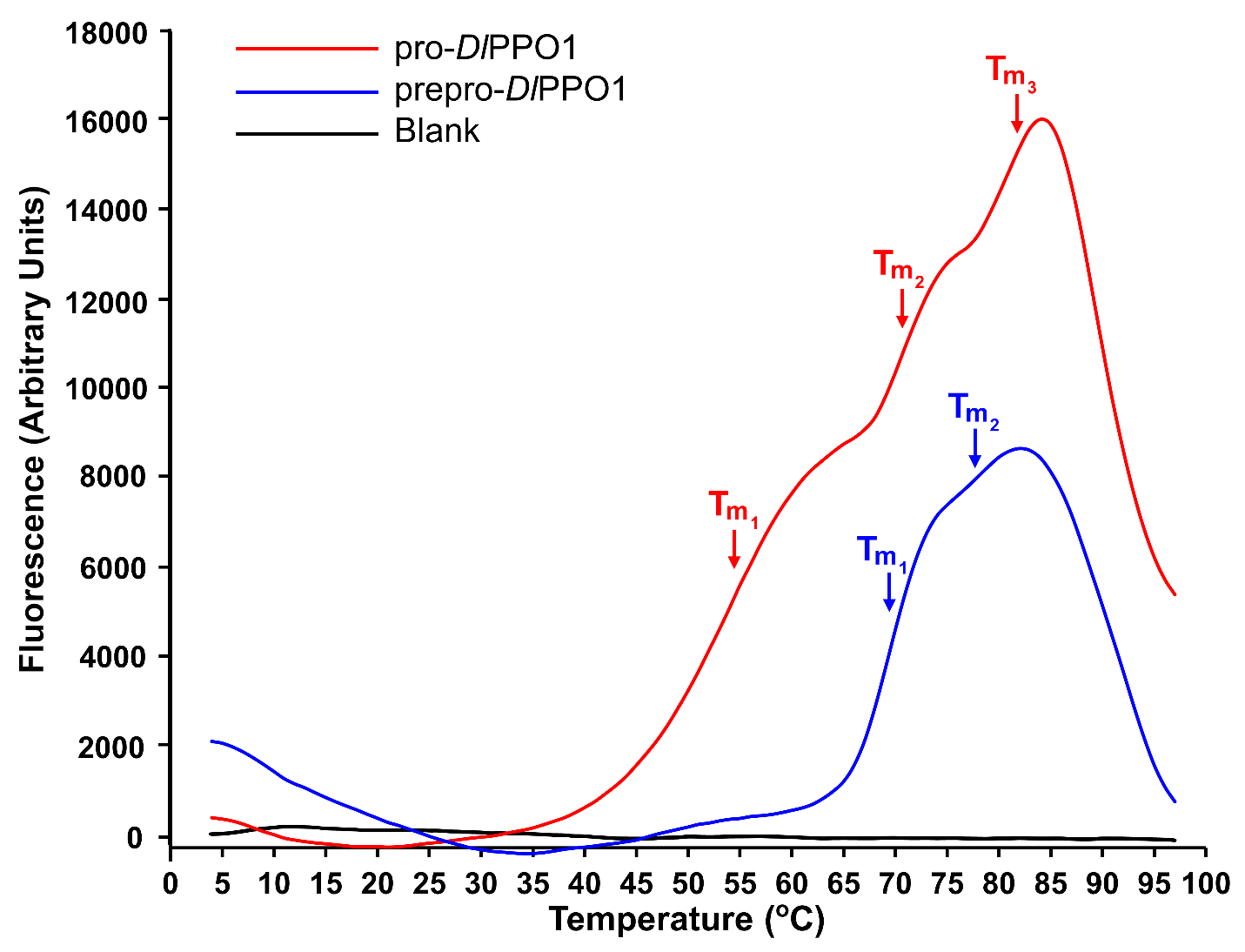


### Figure S9. Fluorescence curves of the thermal shift assay of pro-*Dl*PPO1 and prepro-*Dl*PPO1. The signals are based on the SYPRO Orange dye binding upon thermal unfolding of pro-*Dl*PPO1 (red line) and prepro-*Dl*PPO1 (blue line). The pro-*Dl*PPO1 exhibit melting temperature values (T_m_) of 54.5 °C, 70.5 °C and 81.5 °C while prepro-*Dl*PPO1 showed higher melting temperatures of 69.5 °C and 77.5 °C (Figure 3). The black line shows the control that contains only the buffer with the SYPRO Orange dye without any protein.

###
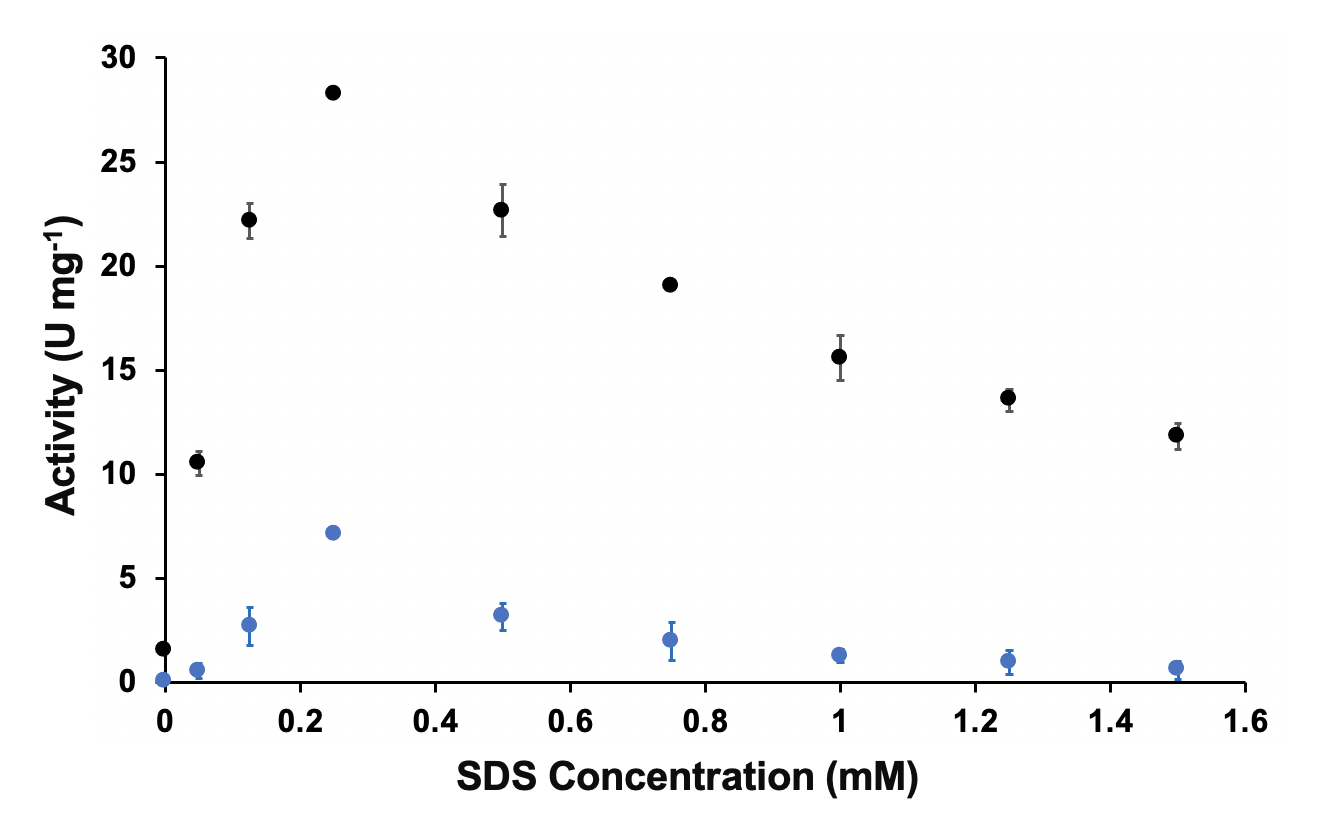


**Figure S10. Effect of SDS concentration on prepro-*Dl*PPO1 and pro-*Dl*PPO1 activities.** Each enzymatic activity was determined using 0.5 µg of the pro-DlPPO1 enzyme (stored in 100 mM MES and 200 mM NaCl pH 6.5) and 8 mM tyramine as substrate in the presence of 50 mM MES buffer pH 7 as the reaction buffer. SDS concentrations were varied as 0.05, 0.1, 0.25, 0.5, 0.75, 1.00, 1.25 and 1.50 mM. The blue data points show specific activity values of prepro-*Dl*PPO1, while the black data correspond to specific activity values of pro-*Dl*PPO1 at the respective SDS concentration. One data point represents the average of three measurements, the error bars show ± one standard deviation.

**
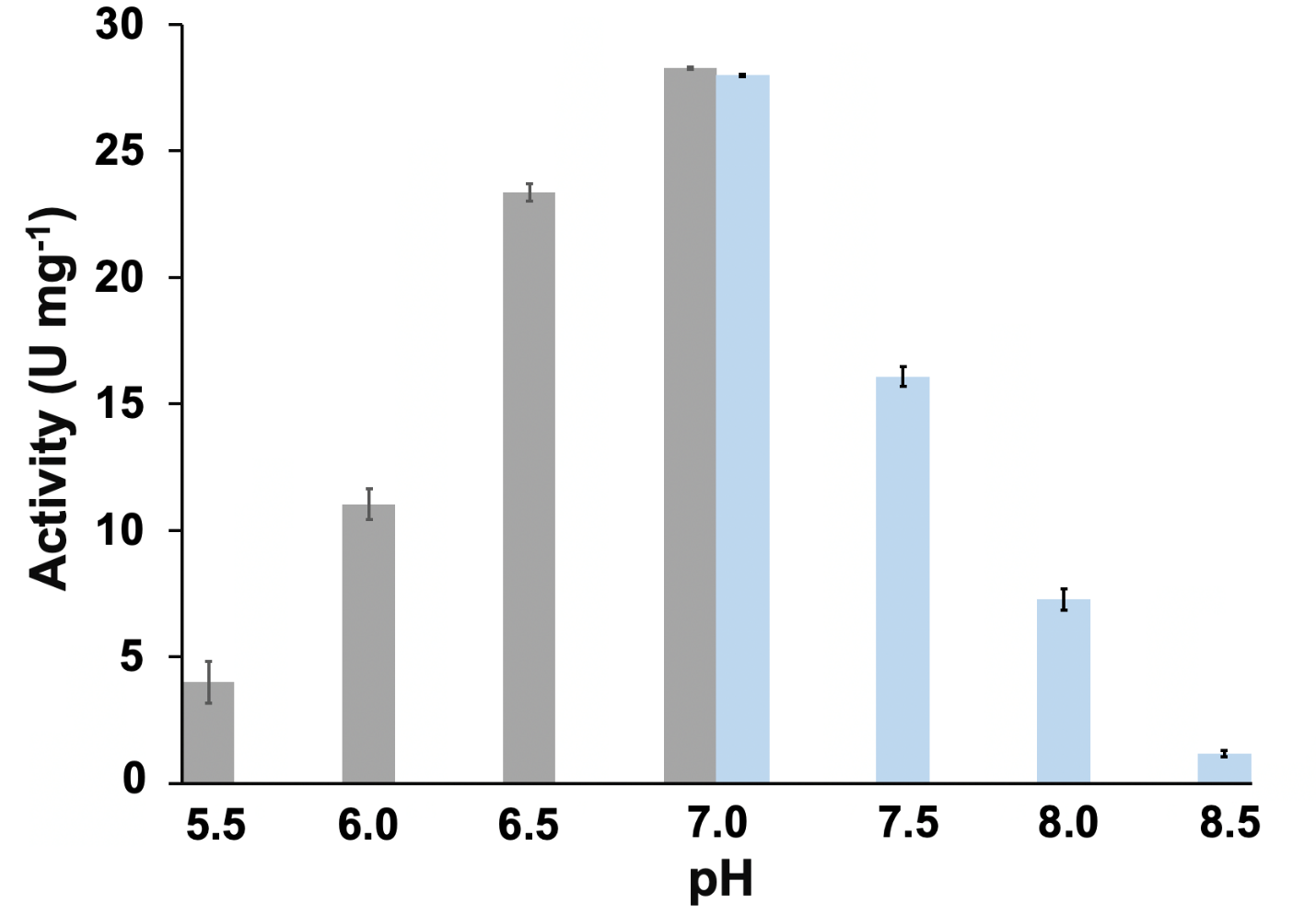
**

**Figure S11. pH-dependence of the reaction rate of SDS-activated pro-*Dl*PPO1 in MES and TRIS buffers.** The assays were carried out using 0.5 µg pro-DlPPO1 (stored in 100 mM MES and 200 mM NaCl pH 6.5), 8 mM tyramine as substrate and 0.25 mM SDS for activation with a series of 50 mM MES (pH 5.5–7.0, shown in grey) and 50 mM Tris buffers (pH 7.0-8.5, shown in blue). One data point represents the average of three measurements, the error bars show ± one standard deviation.

**A)**

**
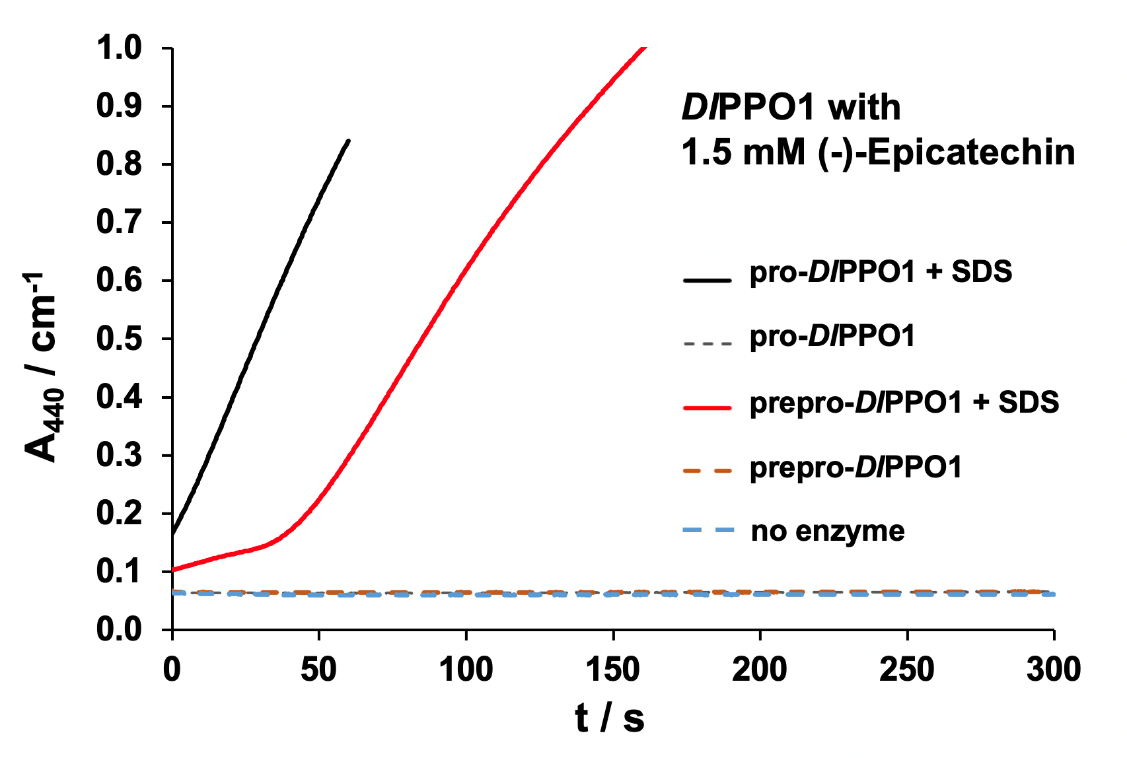
**

**B)**

**
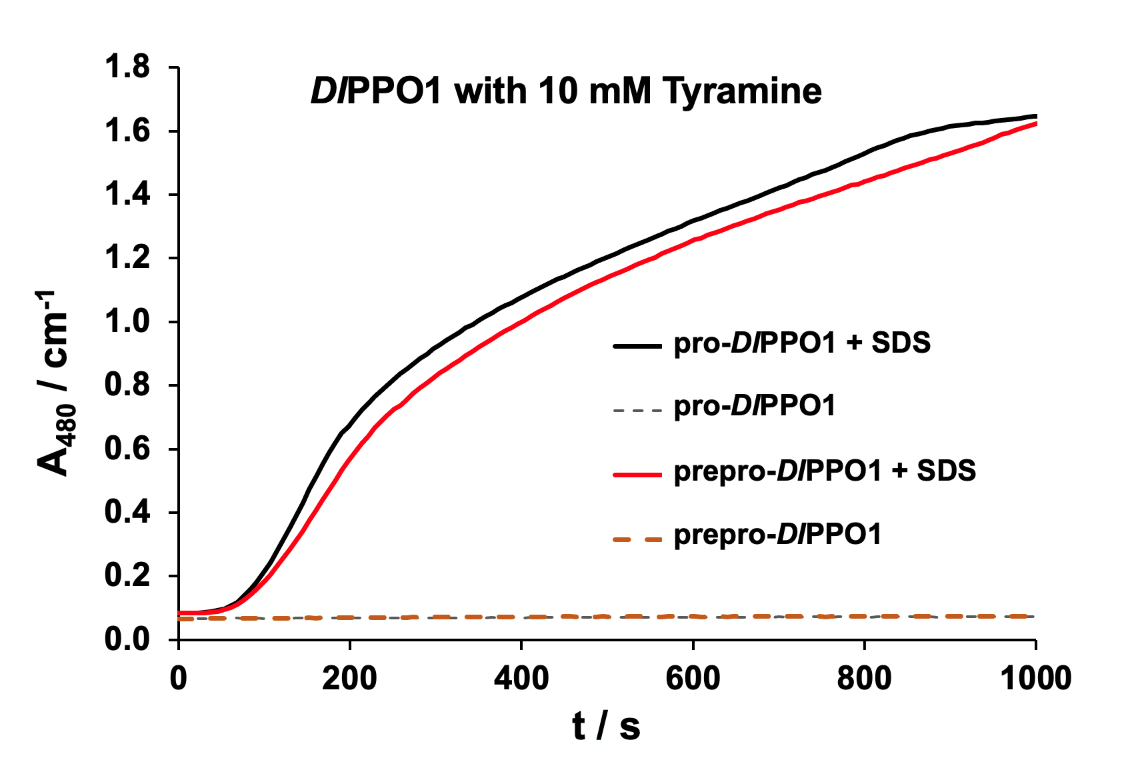
**

**Figure S12. Activity of pro-*Dl*PPO1 and prepro-*Dl*PPO1 without activator.** The assays were carried out with 0.25 µg or 1.3 µg pro-DlPPO1 and 0.26 µg or 1.5 µg prepro-*Dl*PPO1 (both stored in 100 mM MES and 200 mM NaCl pH 6.5) for the tests with **A**) 1.5 mM (-)-epicatechin or **B**) 10 mM tyramine, respectively. Curves named “+ SDS” were recorded with 0.25 mM SDS in the assay solutions, the other curves show the activity without SDS. A 160-fold increase of the used enzyme amount for the reaction with 1.5 mM (-)-epicatechin in the absence of SDS did not result in a significantly different slope of the recorded absorbance-time curve.

**
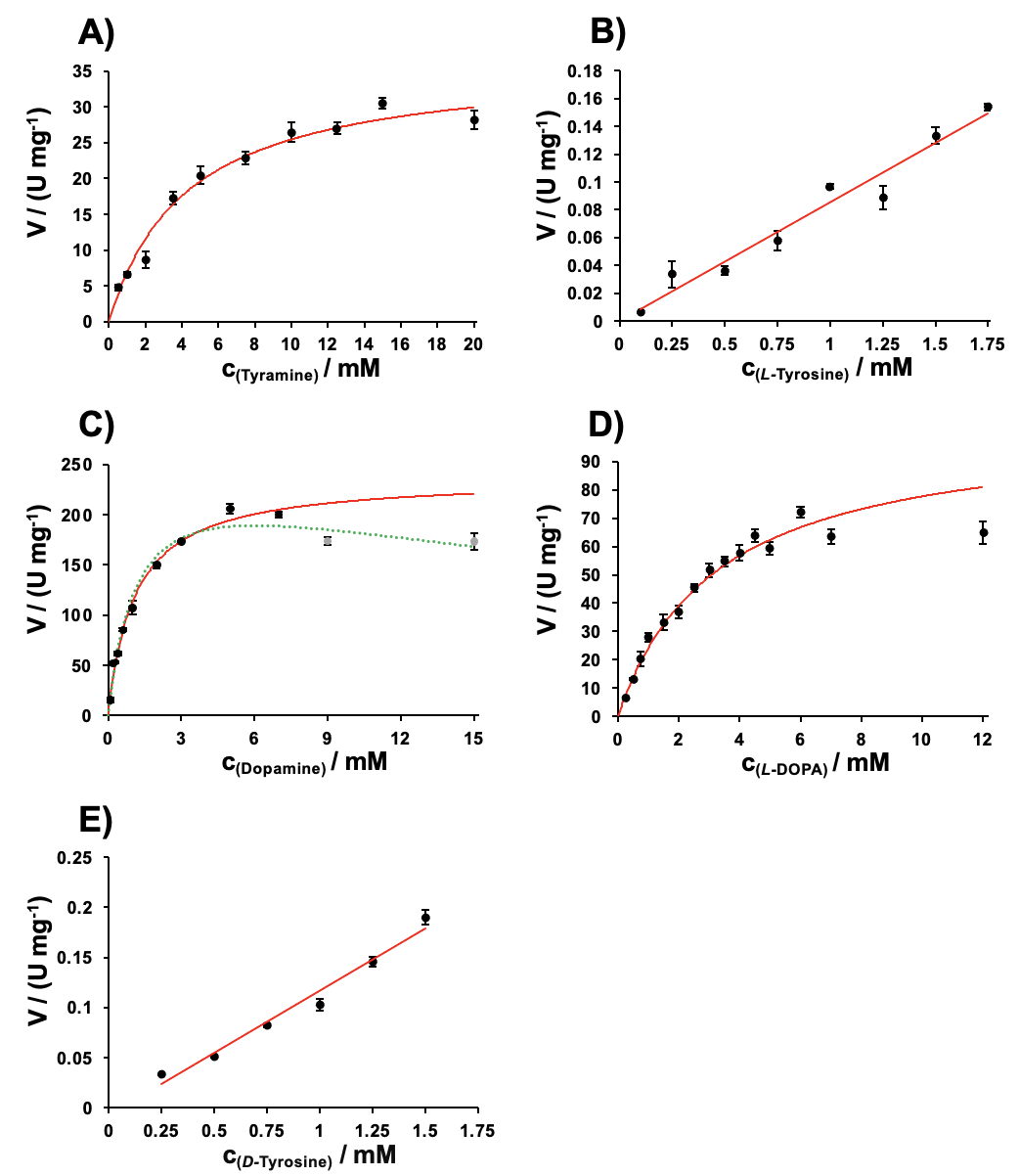
**

**Figure S13. Michaelis-Menten diagrams of the enzymatic reactions of pro-*Dl*PPO1 applying standard phenolic substrates**. **A**) Tyramine, **B**) *l*-Tyrosine, **C**) Dopamine, **D**) *l*-DOPA and **E**) *d*-Tyrosine. Black and light grey data points represent measured slopes. The light grey circles indicate a significant contribution of substrate inhibition, these data points were excluded from data analysis. The resulting Michaelis-Menten models^15^ were obtained as red curves yielding the predicted reaction rates as reported in **Table 3**. The green broken line in **C**) shows a Michaelis-Menten kinetic model with substrate inhibition.^16^ “Standard substrates” refers to common phenolic compounds that are usually used to characterize PPOs but are not reported as constituents of longan fruits.^6-10^

**
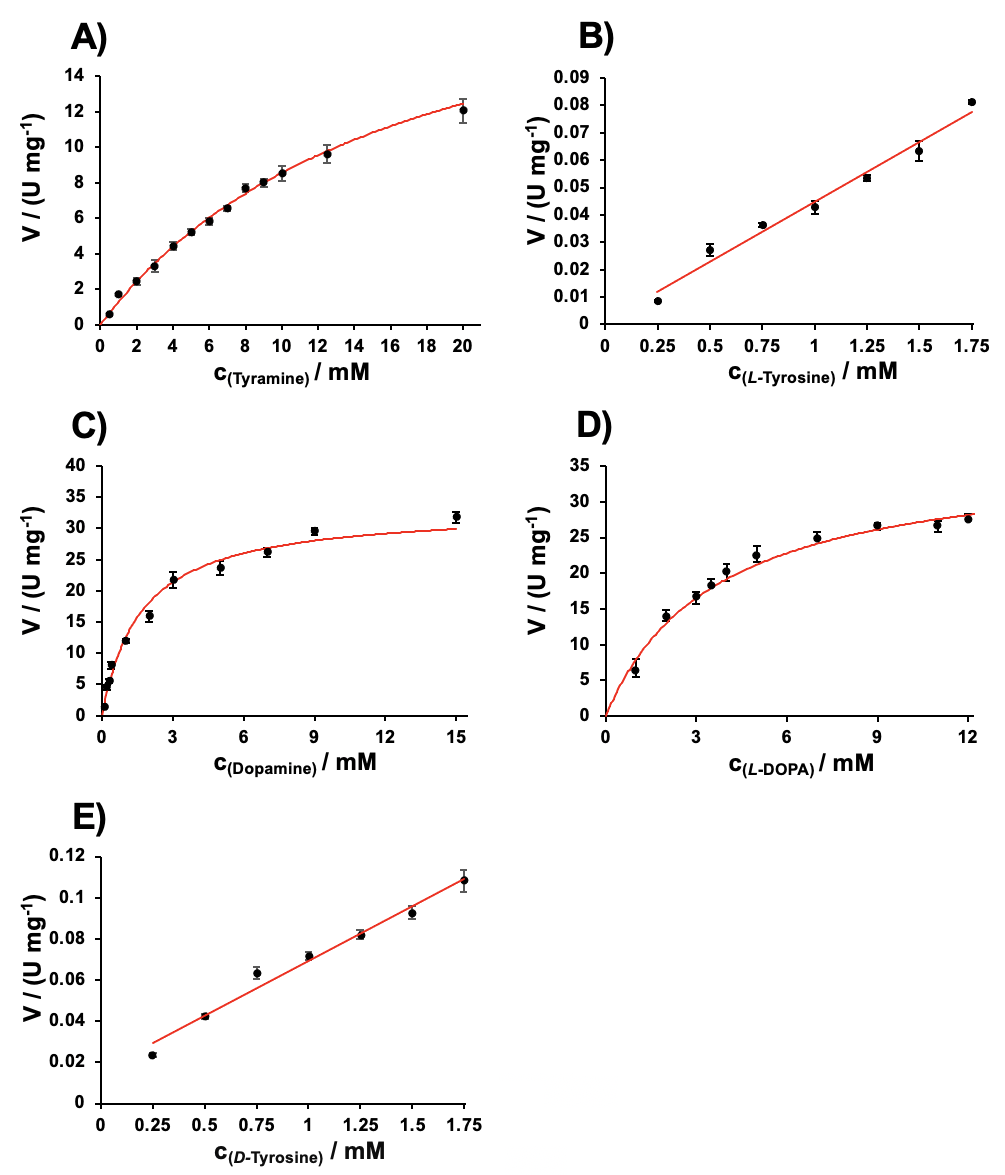
**

**Figure S14. Michaelis-Menten diagrams of the enzymatic reactions of prepro-*Dl*PPO1 applying standard phenolic substrates**. **A**) tyramine, **B**) *l*-tyrosine, **C**) dopamine, **D**) *l*-DOPA and **E**) *d*-tyrosine. Black data points represent measured slopes. The resulting Michaelis-Menten models^15^ were obtained as red curves yielding the predicted reaction rates as reported in **Table 3**. “Standard substrates” refers to common phenolic compounds that are usually used to characterize PPOs but are not reported as constituents of longan fruits.^6-10^

**^
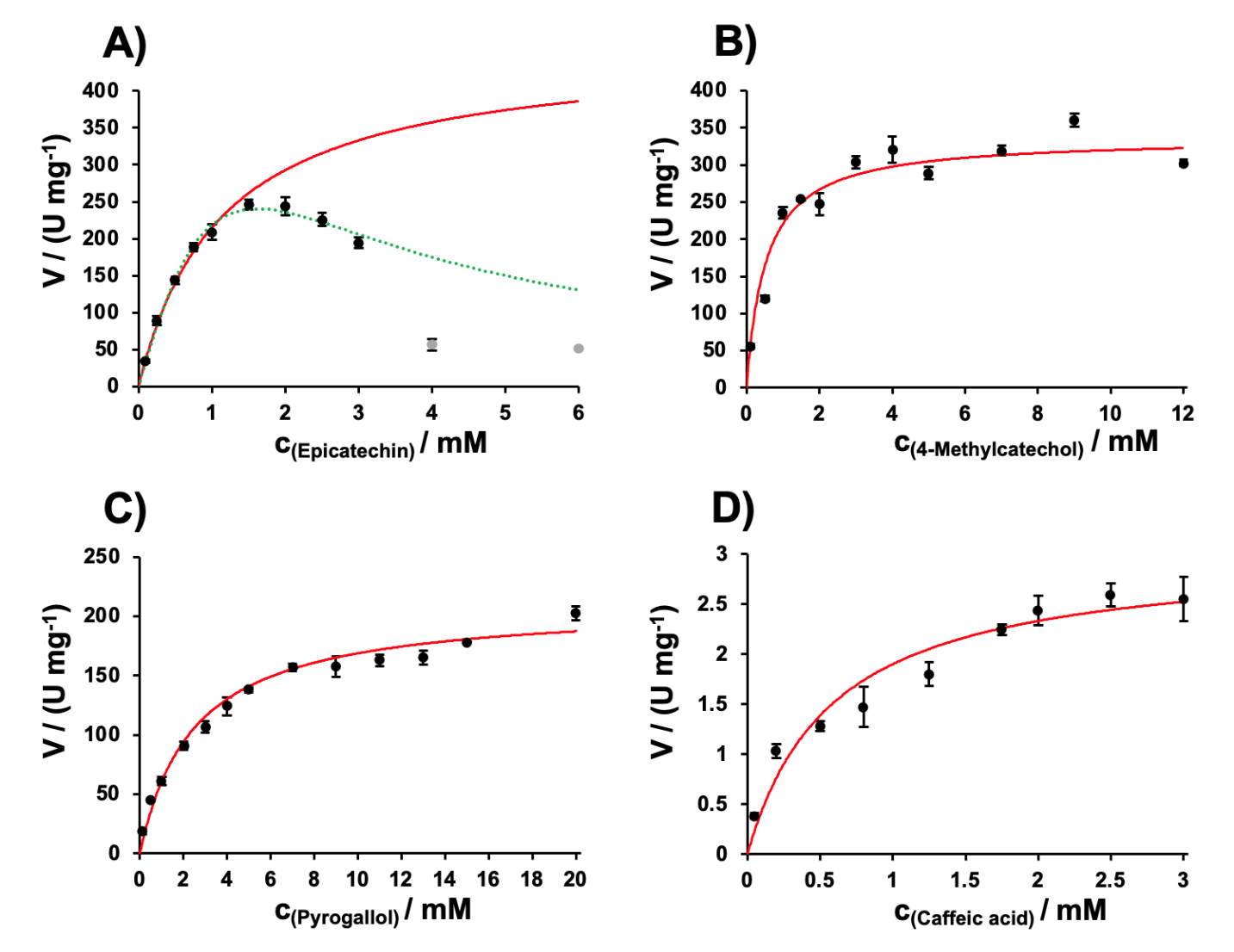
^**

**Figure S15. Michaelis-Menten diagrams of the pro-*Dl*PPO1 enzymatic reactions applying natural phenolic substrates. A**), **B**), **C**) and **D**) represent the fitting curves of the reactions of pro-*Dl*PPO1 with (-)-epicatechin, 4-methylcatechol, pyrogallol and caffeic acid, respectively. Black and light grey data points represent measured slopes. The light grey data circles demonstrate a significant contribution of substrate inhibition and were excluded from data analysis. The resulting Michaelis-Menten models^15^ were obtained as red curves yielding the predicted reaction rates as reported in **Table 3**. The green broken line in **A**) shows a Michaelis-Menten kinetic model with substrate inhibition.^16^ “Natural substrates” refers to phenolic compounds that have been reported to be present in longan fruit.^3-5^

**
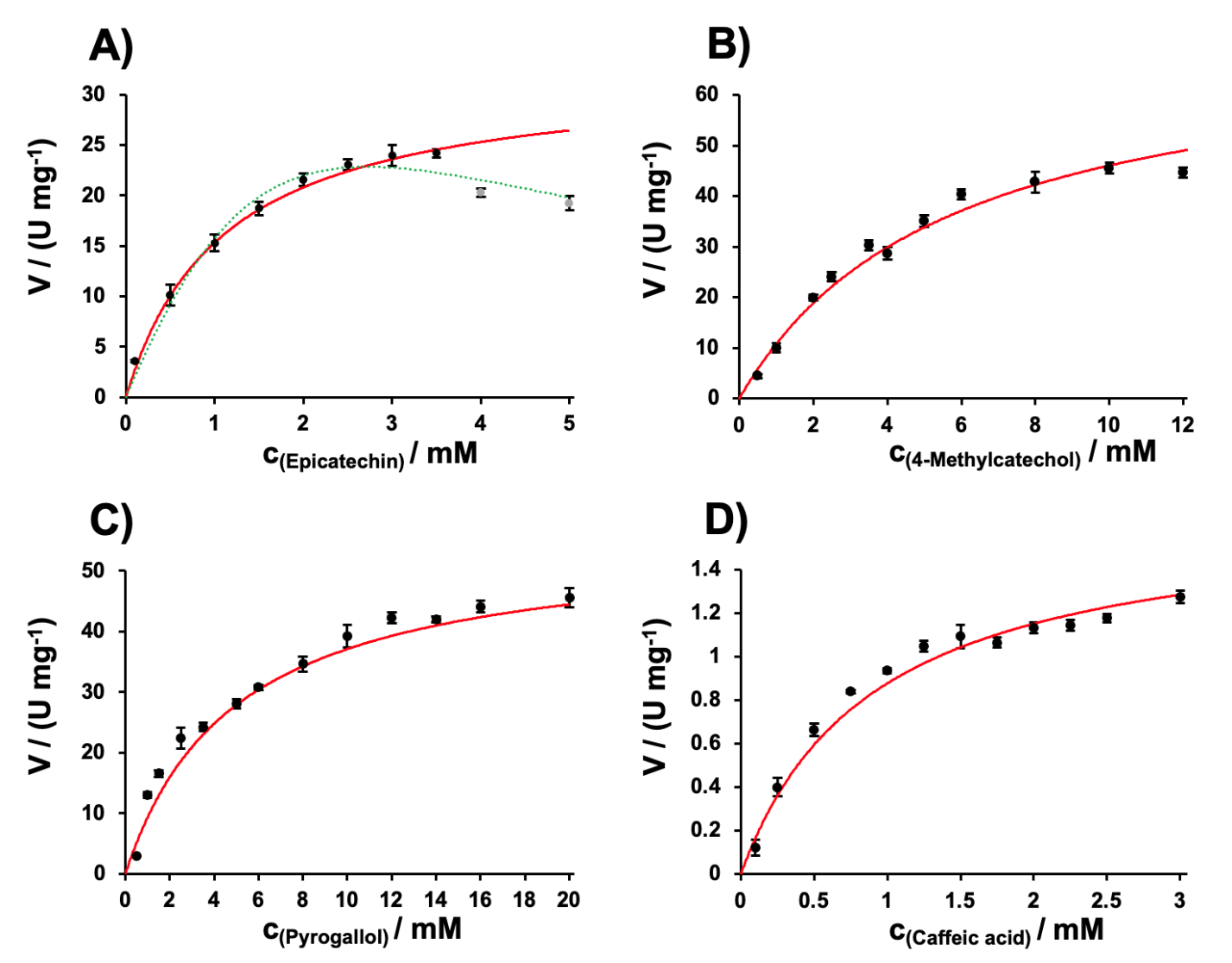
****Figure S16. Michaelis-Menten diagrams of the prepro-*Dl*PPO1 enzymatic reactions applying natural phenolic substrates. A**), **B**), **C**) and **D**) represent the fitting curves of the reactions of prepro-*Dl*PPO1 with (-)-epicatechin, 4-methylcatechol, pyrogallol and caffeic acid, respectively. Black and light grey data points represent measured slopes. The light grey data circles demonstrate a significant contribution of substrate inhibition and were excluded from data analysis. The resulting Michaelis-Menten models^15^ were obtained as red curves yielding the predicted reaction rates as reported in **Table 3**. The green broken line in **A**) shows a Michaelis-Menten kinetic model with substrate inhibition.^16^ “Natural substrates” refers to phenolic compounds that have been reported to be present in longan fruit.^3-5^


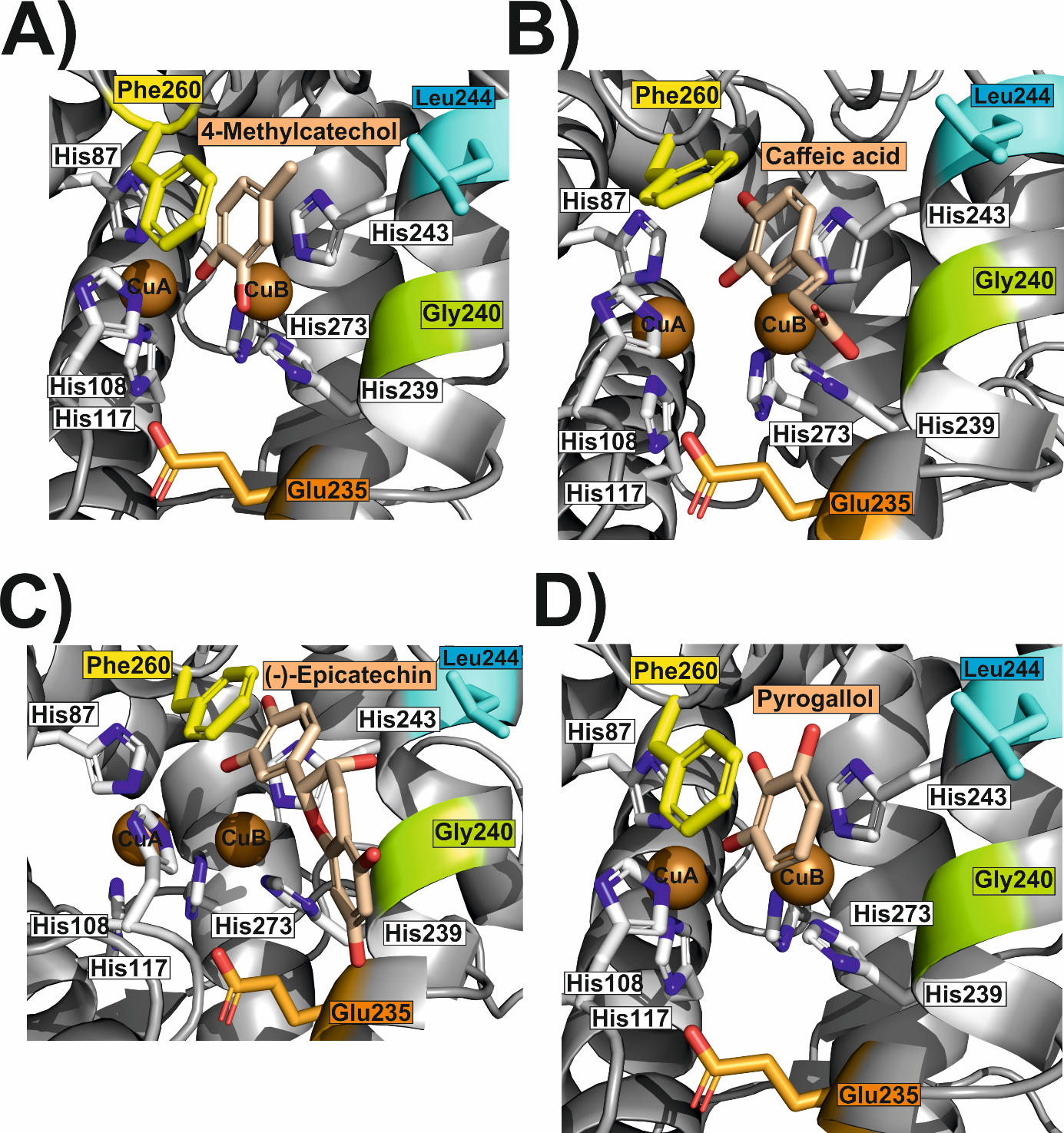


**Figure S17.** **Structural comparison of the interactions between the *Dl*PPO1 active site and the natural substrates obtained from the docking studies. A**) *Dl*PPO1-4-methylcatechol complex, **B**) *Dl*PPO1-caffeic acid complex, **C**) *Dl*PPO1-(-)-epicatechin complex and **D**) *Dl*PPO1-pyrogallol complex. Detailed information regarding the calculation setup is provided in the Materials and Methods section. The interactions of the *Dl*PPO1 active site with gallic acid, quercetin, vanillic acid and ferulic acid were excluded from the docking studies due to the absence of activity of *Dl*PPO1 towards these substrates observed in the kinetic experiments (**Table 3**). “Natural substrates” refers to phenolic compounds that have been reported to be present in longan fruit.^3-5^


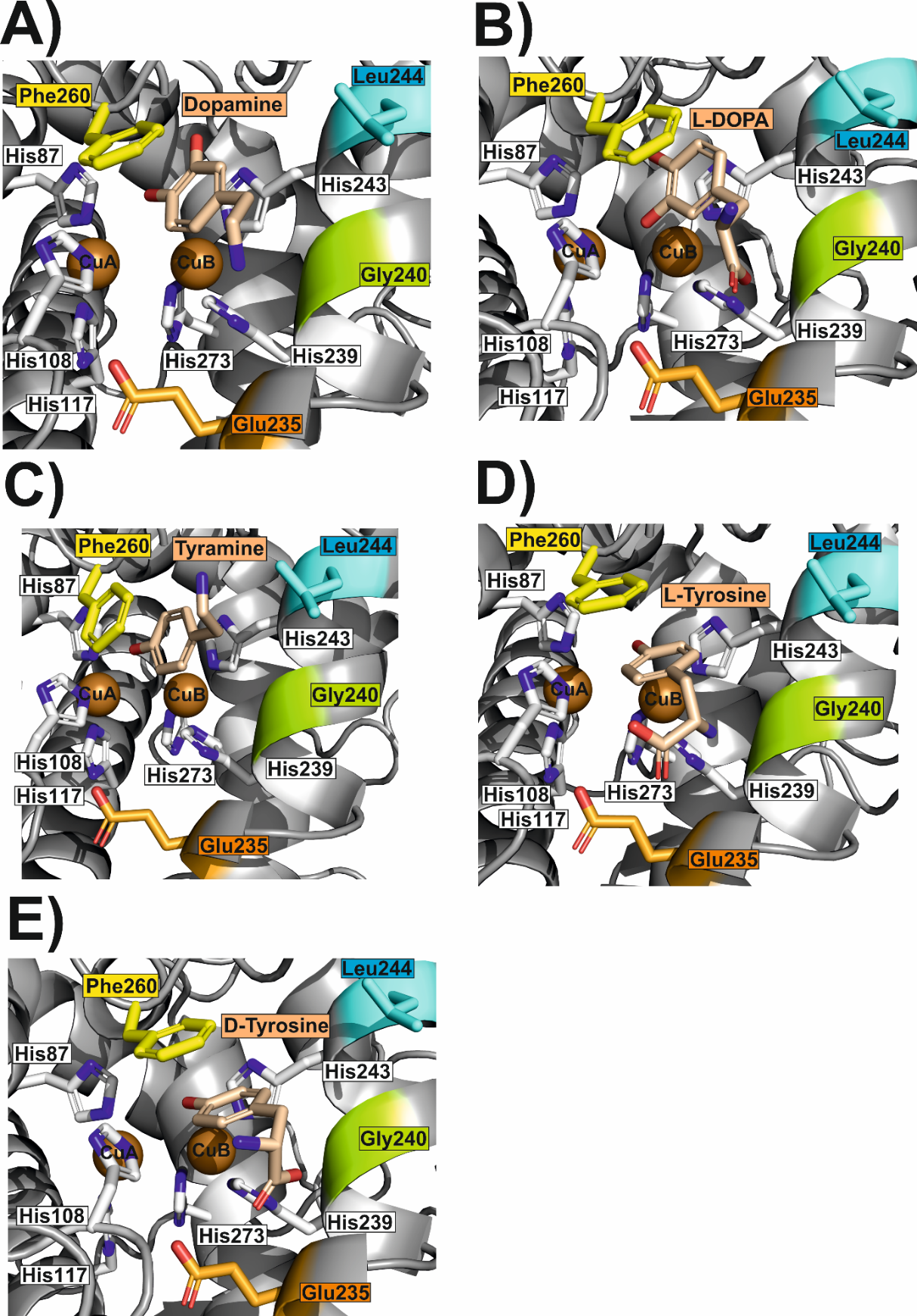


**Figure S18. Structural comparison of the interactions between the *Dl*PPO1 active site and the standard substrates obtained from docking studies.** **A**) *Dl*PPO1-dopamine complex, **B**) *Dl*PPO1-*l*-DOPA complex, **C**) *Dl*PPO1-tyramine complex, **D**) *Dl*PPO1-*l*-tyrosine complex and **E**) *Dl*PPO1-*d*-tyrosine complex. Detailed information regarding the calculation setup is provided in the Materials and Methods section. “Standard substrates” refers to common phenolic compounds that are usually used to characterize PPOs but are not reported as constituents of longan fruits.^6-10^

**
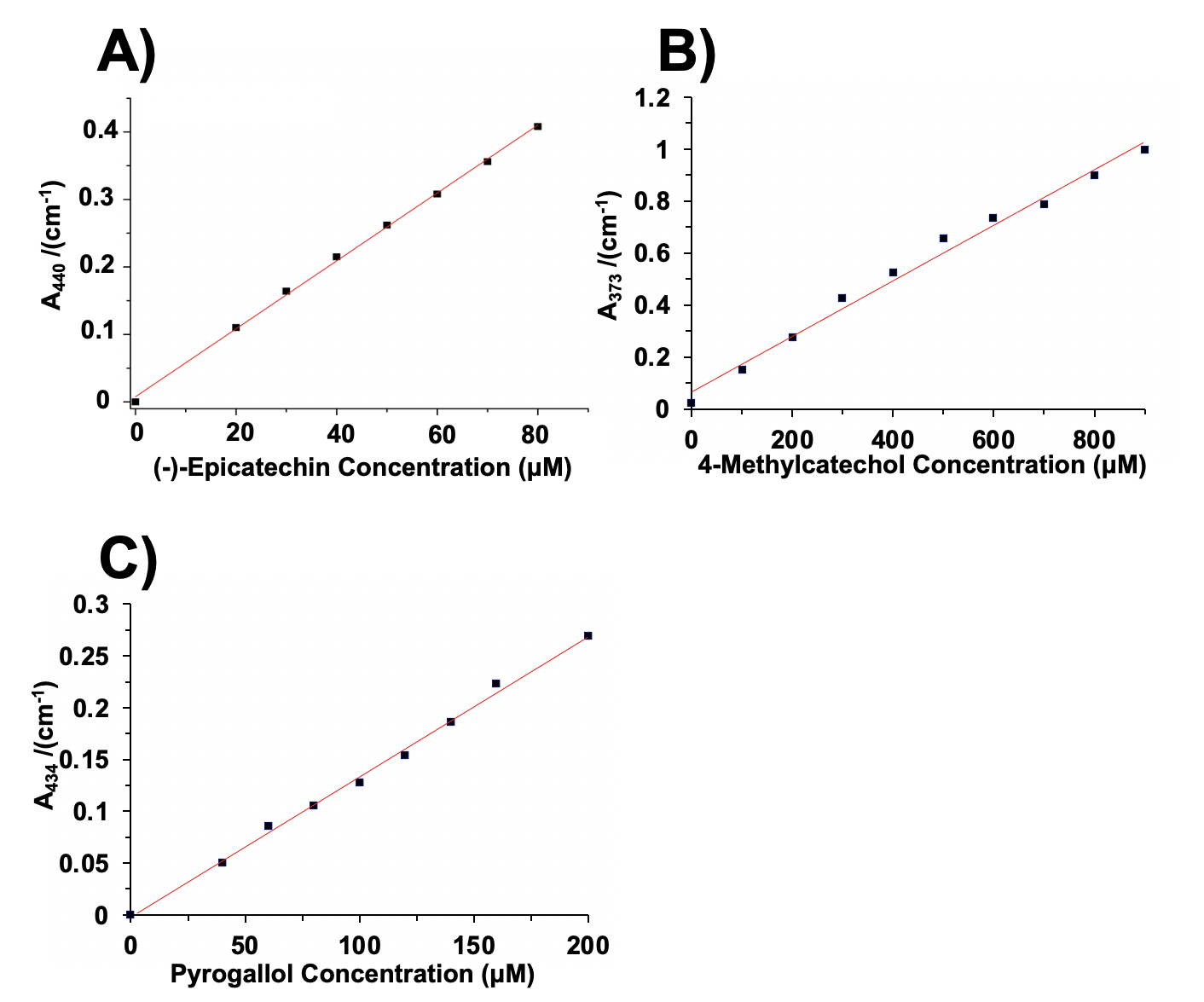
**

**Figure S19. Absorbances obtained by oxidation of phenolic substrates to determine the molar extinction coefficients using linear curve fitting.** The substrates **A**) (–)-Epicatechin (440 nm), **B**) 4-Methylcatechol (373 nm) and **C**) Pyrogallol (434 nm) were measured in 50 mM MES buffer at pH 7 after oxidation with sodium periodate^11^. Regression lines used to calculate the molar extinction coefficients were calculated by linear curve fitting. The resulting molecular extinction coefficients are listed in **Table S4**.

### 4. Equations

$V=\frac{V_{max}\cdot\left[ S \right]}{K_{M}+\left[ S \right]}$ **Equation S1**

*V* rate of enzymatic reaction, in mol l^-1^ s^-1^ = M s^-1^

*V_max_* maximal rate of the enzymatic conversion, in M s^-1^

*[S]* substrate concentration, in mol l^-1^ = M

*K_M_* Michaelis constant; substrate concentration for V = 0.5 V_max_, in M

$k_{cat}=\frac{V_{max}}{\left[ E \right]_{0}}$ **Equation S2**

*k_cat_* enzymatic turnover rate; converted substrate molecules per enzyme, in s^-1^

[*E*]_0_ (initial) enzyme concentration, in M

$V=\frac{V_{max}\cdot\left[ S \right]}{K_{M}+\left[ S \right]+\frac{\left[ S \right]^{2}}{K_{i}}}=\frac{V_{max}}{\frac{K_{M}}{\left[ S \right]}+1+\frac{\left[ S \right]}{K_{i}}}$ **Equation S3**

*K_i_* substrate inhibition constant, in M

**5. References**

1. Arita, M., Karsch-Mizrachi, I. & Cochrane, G. On behalf of the International Nucleotide Sequence Database Collaboration, The international nucleotide sequence database collaboration. *Nucleic Acids Research*, **49**, D1, 121-124 (2021). doi: [10.1093/nar/gkaa967](https://doi.org/10.1093/nar/gkaa967)
2. Meija, J., Coplen, T. B., Berglund, M., Brand, W. A., De Bièvre, P., Gröning, M., Holden, N. E., Irrgeher, J., Loss, R. D., Walczyk, T. & Prohaska, T. Isotopic compositions of the elements 2013 (IUPAC Technical Report). *Pure and Applied Chemistry* **88**, 293–306 (2016). doi: [10.1515/pac-2015-0503](https://doi.org/10.1515/pac-2015-0503)
3. Rangkadilok, N., Worasuttayangkurn, L., Bennett, R. B. & Satayavivad, J. Identification and Quantification of Polyphenolic Compounds in Longan (*Euphoria longana* Lam.) Fruit. *J. Agric. Food Chem.* **53**, 1387–1392 (2005). doi: [10.1021/jf0403484](https://doi.org/10.1021/jf0403484)
4. He, N. *et al.* Isolation and identification of polyphenolic compounds in longan pericarp. *Sep. Purfication Technol.* **70**, 219–224 (2009). doi: [10.1016/j.seppur.2009.09.019](https://doi.org/10.1016/j.seppur.2009.09.019)
5. Zhang, R., Khan, S. A., Lin, Y., Guo, D. & Pan, X. Phenolic profiles and cellular antioxidant activity of longan pulp of 24 representative Chinese cultivars. *Int. J. Food Prop.* **21**, 746–759 (2018). doi: [10.1080/10942912.2018.1425705](https://doi.org/10.1080/10942912.2018.1425705)
6. Pretzler, M., Bijelic, A. & Rompel, A. Heterologous expression and characterization of functional mushroom tyrosinase (*Ab*PPO4). *Sci. Rep.* **7**, 1810 (2017). doi: [10.1038/s41598-017-01813-1](https://doi.org/10.1038/s41598-017-01813-1)
7. Kampatsikas, I., Bijelic, A. & Rompel, A. Biochemical and structural characterization of tomato polyphenol oxidases provide novel insights into their substrate specificity. *Sci. Rep.* **9**, 4022 (2019). doi: [10.1038/s41598-019-39687-0](https://doi.org/10.1038/s41598-019-39687-0)
8. Kampatsikas, I., Bijelic, A., Pretzler, M. & Rompel, A. Three recombinantly expressed apple tyrosinases suggest the amino acids responsible for mono- versus diphenolase activity in plant polyphenol oxidases. *Sci. Rep.* **7**, 8860 (2017). doi: [10.1038/s41598-017-08097-5](https://doi.org/10.1038/s41598-017-08097-5)
9. Panis, F. & Rompel, A. Identification of the amino acid position controlling the different enzymatic activities in walnut tyrosinase isoenzymes (*jr*PPO1 and *jr*PPO2). *Sci. Rep.* **10**, 10813 (2020). doi: [10.1038/s41598-020-67415-6](https://doi.org/10.1038/s41598-020-67415-6)
10. Panis, F., Krachler, R. F., Krachler, R. & Rompel, A. Expression, Purification, and Characterization of a Well-Adapted Tyrosinase from Peatlands Identified by Partial Community Analysis. *Environ. Sci. Technol.* **55**, 11445-11454 (2021). doi: [10.1021/acs.est.1c02514](https://doi.org/10.1021/acs.est.1c02514)
11. Muñoz, J. L. *et al.* Calculating molar absorptivities for quinones: Application to the measurement of tyrosinase activity. *Anal. Biochem.* **351**, 128–138 (2006). doi: [10.1016/j.ab.2006.01.011](https://doi.org/10.1016/j.ab.2006.01.011)
12. Kampatsikas, I., Bijelic, A., Pretzler, M. & Rompel, A. A Peptide-Induced Self-Cleavage Reaction Initiates the Activation of Tyrosinase. *Angew. Chemie - Int. Ed.* **58**, 7475–7479 (2019). doi: [10.1002/anie.201901332](https://doi.org/10.1002/anie.201901332)
13. Qilin, T., Yuling, L., Qingyou, Z., Rongfeng, S. & Zhongxiong, L. Cloning and expression analyses of *Dl*PPO1 from *Dimocarpus Longan Lour*. *Acta Bot. Boreal.-Occid. Sin.* **36**, 1098–1104 (2016).
14. Derardja, A., Pretzler, M., Kampatsikas, I., Barkat, M. & Rompel, A. Purification and Characterization of Latent Polyphenol Oxidase from Apricot (*Prunus armeniaca* L.). *J. Agric. Food Chem.* **65**, 8203–8212 (2017). doi: [10.1021/acs.jafc.7b03210](https://doi.org/10.1021/acs.jafc.7b03210)
15. Michaelis, L. & Menten, M.L. Die Kinetik der Invertinwirkung. *Biochemische Zeitschrift* **49**, 333-336 (1913).
16. Reed, M. C., Lieb, A. & Nijhout, H. F. The biological significance of substrate inhibition: A mechanism with diverse functions *BioEssays* **32**, 422-429 (2010). doi: [10.1002/bies.200900167](https://doi.org/10.1002/bies.200900167)
